# Classical Morphometrics in *V. arvensis* and *V. kitaibeliana* (*Viola* sect. *Melanium*) Reveals Intraspecific Variation with Implications for Species Delimitation: Inferences from a Case Study in Central Italy

**DOI:** 10.3390/plants11030379

**Published:** 2022-01-29

**Authors:** Anna Scoppola, Daniele Angeloni, Cinzia Franceschini

**Affiliations:** 1 Department of Agricultural and Forestry Sciences (DAFNE), Tuscia University, Via S. Camillo de Lellis, 01100 Viterbo, Italy; angelonidaniele94@gmail.com; 2 Department of Biological, Geological, and Environmental Sciences (BIGeA), University of Bologna, Piazza di Porta S. Donato, 40127 Bologna, Italy; cinziafranceschini@msn.com

**Keywords:** *Viola* sect. *Melanium*, morphological variation, linear discriminant analysis, joint relative frequencies, eco-phenotype, Central Italy

## Abstract

The high morphological variability of *Viola arvensis* may hinder the proper identification of the closely related species with an implication for biodiversity surveys. Variation in floral and vegetative morphology was explored in *V. arvensis*, compared to *V. kitaibeliana*, based upon 14 wild Italian populations, to provide new insights into their diagnostic features. Species were characterized using 32 morphological descriptors assessed on 272 flowers and as many leaves and scored as quantitative and categorical variables. Statistical methods, including Linear Discriminant Analysis (LDA), were applied to test species delimitation. Data highlighted variations in sepal size, petal size, leaves shape, stylar dark spot, and pollen magazine higher within *V. arvensis* than between *V. arvensis* and *V. kitaibeliana*. LDA partitioned the *V. arvensis* samples into two distinct clusters; no clear distinction was found between the cluster combining individuals from grasslands and *V. kitaibeliana*. The separation of *V. arvensis* and *V. kitaibeliana* from *V. tricolor*, included as a reference, was noticeable. Correlations were found in all species between the flower/leaf position on the stem and some floral and vegetative features. The shape and margin of the lower sepal, the stylar flap, and the lamina margin and apex were diagnostic in field identification. The results support the recognition of an undescribed *V. arvensis* eco-phenotype linked to seminatural dry grasslands, easily distinguishable from the field-grown type of *V. arvensis* but hardly distinguishable from the dwarf pansy. Data further corroborate the assumption of general deep-rooted confusion in ascribing poorly developed individuals of *V. arvensis* to the rare and locally threatened *V. kitaibeliana*, leading to potential implications for its conservation.

## 1. Introduction

Despite the increase of molecular studies, comparative morphology remains a key tool in plant species distinction, helpful in selecting characters to be used in visual identification [1,2,3,4]. In studies concerning the section *Melanium* DC. ex Ging. of the genus *Viola* L., comparative morphology provided significant insight into systematics, reproductive strategies, and ecological adaptation, as recently highlighted (e.g., [5,6,7,8,9,10]). On the other hand, it was not satisfactory when used alone for delimiting some critical taxa of the genus, e.g., *V. odorata* L. [11] or *V. suavis* M. Bieb. [12]. Therefore, a sound description of the morphological variants to be investigated is a necessary step towards a correct (i.e., well-targeted) application of advanced molecular tools.

*Viola* sect. *Melanium* is a derived and morphologically well-defined section comprising perennial and annual pansies [13] from a wide range of natural and agricultural habitats. Its distribution range includes westernmost Asia and Europe, with a centre of diversity on the Southern Europe hills and mountains, especially the Balkan Peninsula and the Apennines [14]. This section’s annual or short-lived biennial pansies are represented in Europe by some 20 species. They are mostly related to *Viola tricolor* L. (2*n* = 26, [15]), a European temperate element [16] rare in Southern Europe, where it grows typically in deep, sub-acid soils, and habitats linked to woodland [14,17,18]. A single species belonging to the *V. tricolor* species complex is native to NE-America, *Viola rafinesquei* Greene (syn. *V. bicolor* Pursh) [19,20,21] which has never been reported in Eurasia [18]. Annual pansies are weeds in nature, growing under disturbed conditions (i.e., rural and human-made habitats) and rarely occurring in undisturbed habitats [14,19].

Variously branched stems can distinguish the *V. tricolor* species complex, with alternate leaf arrangement, leaves crenate-dentate or shallowly crenate to entire, leaf-like stipules deeply divided and varying from pinnately lobed to palmately lobed [7,8,14]. Flowers are chasmogamous, solitary on long peduncles arising from the leaves’ axil. They consist of sepals ovate-lanceolate to narrowly lanceolate-acute, auricled at the base, corolla frontally flattened with two upper petals, two upwards turned side petals, and a lower petal with a honeyguide of variable length on the proximal part, and a scarcely exerted spur at the back. The pollen assemblage and the pollen morph vary by species [10]. The stigma is capitate with or without a ventral stylar flap just under the orifice to the stigmatic chamber, clearly appearing downturned in prevailing inbreeder pansies and forward turned in outbreeder [5,7,22]. The fruit is a multi-seeded capsule.

Authors’ different species concepts and circumscriptions to this group have contributed to the large number of taxa described in Europe during the last two centuries [13,23,24,25,26]. Authors [8,14] pointed out that proper identification of such related species requires experience and the analysis of a significant number of macro- and micro-morphological features. The reason is the high uniformity in the floral structure appearance combined with seasonal dimorphism and high intra-specific variability in the vegetative features [7]. Species boundaries and relationships in the *V. tricolor* species complex are not fully understood, despite a recent wide usage of molecular characterizations alongside morphometrics in related species (e.g., [8,27,28,29]). Referring to the five annual pansies recorded in Italian flora, we have previously stressed the difficulty in labelling a given sample on purely macro-morphological criteria [7], assuming the presence of intermediate forms due to frequent introgression and interspecies hybridization. Hybrids are already known among almost all the species of the section in their European distribution areas [8,14,30,31,32,33,34,35]. In these cases, as in extreme morphological forms and poorly developed specimens, the chromosome number would appear an essential species-delimiting attribute [23,26,36]. Recent authors [10] have highlighted the seed size and seed coat micro-morphology as additional distinguishing characters, particularly useful in specimens bearing capsules with mature seeds when other diagnostic characters are missing or inconspicuous.

*V. arvensis* Murray (2*n* = 34, [15]) (field pansy) is regarded as a weed requiring ephemeral habitats commonly occurring in arable and waste ground, fields, open scrublands, and other agricultural and ruderal places [17,35,37,38]. Weedy nature does not prove that it is an alien plant throughout its range [20]. It is currently considered native to SE-Europe and W-Asia, naturalised throughout the rest of Europe, with a worldwide secondary distribution in the temperate zones of the northern and southern hemispheres [17,37,39,40]. The field pansy is reported as invasive in 12 countries or islands, including USA, Australia, and in Argentina where there is evidence of impact [37]. *V. kitaibeliana* Schult. (2*n* = 16, [41,42]) (dwarf pansy) is a winter annual component of early stages of grasslands, stony slopes, and screes on calcareous soil, more rarely of fallow land, banks, and other open places in rural environments [7,14,28,43]. The dwarf pansy is a Mediterranean-Caucasian species endemic to the Med-Checklist area [44], extending to Central Europe. It has a highly fragmented distribution in Italy. The two species have intertwined nomenclatural histories; at various times and until the beginning of the twentieth century, they were commonly regarded as varieties of *V. tricolor* [7,19,36] or associated with each other [45,46]. The high phenotypical plasticity of *V. arvensis* [47,48] and the hypothesized overlapping variation of the features traditionally assigned to both species are the most likely obstacles to their entirely resolved taxonomy. In *V. kitaibeliana*, two infraspecific taxa are currently accepted, *V. kitaibeliana* subsp. *trimestris* (DC. ex Ging.) Espeut from SW Europe, and *V. kitaibeliana* subsp. *machadiana* Capelo and C. Aguiar, native to Portugal [21]. In the European distribution area of *Viola arvensis*, only *V. arvensis* subsp. *megalantha* Nauenb., native to Albania, Austria, Italy, and Switzerland, is currently accepted [16,26], though many other local varieties and forms have been reported based on combinations of floral and vegetative characters and distribution range [16,26]. They all are treated as synonyms in the principal taxonomic databases (e.g., [49,50,51]). *V. arvensis* from shallow and poor soils has often been confused with *V. kitaibeliana* (e.g., [7,17,52]. The lack of observable features on the type material of *V. kitaibeliana* (Lectotype: In Pannonia, Kitaibel, M0112803) makes it difficult to compare the two species. *V. arvensis* on deep and fertilized soils or forest edges could also be confused with *V. tricolor* s. s. due to broader leaves and more developed flowers [7,25]. To the best of our knowledge, no in-depth morphometric statistical analyses on *V. arvensis* and *V. kitaibeliana* have been done yet. Furthermore, little information deduced from measured values is currently available on *V. arvensis* intraspecific variation (e.g., [47]).

The taxa involved in the study were *Viola arvensis* subsp. *arvensis*, hereafter called *V. arvensis* (*Va*), and *Viola kitaibeliana* subsp. *kitaibeliana*, hereafter called *V. kitaibeliana* (*Vk*). The study did not deal with *V. hymettia* Boiss. and Heldr. and *V. parvula* Tineo, two Italian annual pansies morphologically well delimited [7,14,17,53,54]. We wanted to check whether intraspecific morphological variation in *V. arvensis* could be higher than variation between *V. arvensis* and *V. kitaibeliana*. The aims of the study were: (1) to analyse a broad set of morphological features in the flowers and leaves of the target species and in *Viola tricolor* subsp. *tricolor* (hereafter indicated as *V. tricolor* or *Vt*) as a reference species, considering the single measured values in addition to the average values per population; (2) to provide valuable combinations of characters to circumscribe better and define populations of *V. arvensis* from different habitats and eventually highlight morphological variants therein; (3) to provide discrimination between *V. arvensis* and *V. kitaibeliana* potentially occurring in the same habitats.

The topic of this study is especially relevant as it contributes to strengthening the current understanding of the identity and delimitation of *V. kitaibeliana*, the possibly more ancient lineage in the *V. tricolor* group cf. [19], providing basic data, thus supporting the conservation strategies for a rare and controversial species of European flora.

## 2. Results

### 2.1. Descriptive Statistics and One-Way ANOVA

Correlations among the quantitative variables are provided as Supplementary Material (Table S1). As expected, most floral variables, especially lower petal length (LOP_LE) and spur length (SP_LE), are highly and positively correlated (r > 0.80) to each other. As for vegetative variables, positive and highly significant correlations are reached in the following pairs: lamina length (LA_LE) vs. lamina width (LA_WI), lamina length and width vs. peduncle length (PED_LE), petiole length (PET_LE) vs. lamina width, and stipule length (ST_LE) vs. almost all the above-cited variables. No noticeable correlations are detected by considering floral and vegetative variables in pairs, except for lower sepal length (SE_LE) vs. peduncle length, vs. lamina length, and vs. stipule length (ST_LE), and corolla length (CO_LE) vs. lamina length (r > 0.80). Half lamina teeth number (HALA_TE) and stipule external lobes number (ST_EXLO) do not have a high correlation with the studied variables (Table S1).

Average values (means ± SD, minimum and maximum values) for variables and indices per population are provided as Supplementary Material (Table S2a–c). They highlight an overall morphological uniformity in the mean size of characters within *Vk*. Corolla mean values ranges from 4.94 ± 2.50 mm to 6.29 ± 1.62 mm in length, from 4.02 ± 2.10 mm to 5.52 ± 1.20 mm in width. The lower petal length ranges from 7.07 ± 0.57 mm to 7.31 ± 0.60 mm, and the lamina length from 3.71 ± 0.65 mm to 7.95 ± 2.66 mm. Categorical variables in the three *Vk* populations show the same homogeneous features but lower sepal appendage, both entire (prevailing) and irregularly sinuate, and stipule midlobe margin, both entire and crenate-dentate. Conversely, in *Va*, data concerning both the vegetative (e.g., lamina size, petiole and stipules length, stipule midlobe margin and shape, peduncle length) and the floral characters (e.g., corolla length and width, lower petal and spur length, lower sepal length) show a broad amount of variability among populations. A remarkable likeness emerges within two groups of *Va* populations: group 1 comprising VA-ST, VA-LA, VA-SCIs, VA-TA, VA-CE, showing weak plants with smaller flowers, and group 2 comprising VA-B/1, VA-B/2, VA-BS, VA-AZ, VA-SG, larger in all features. In this second group, VA-AZ is the population with the smallest flowers (CO_LE 10.39 ± 1.70 mm) and petals entirely included in the calyx (LOP/SE_LE 0.97 ± 0.27); larger flowers, distinctly exceeding the calyx, are in VA-B/2 (CO_LE 16.34 ± 2.18 mm, LOP/SE_LE 1.45 ± 0.12), whereas all other populations are intermediate. Group 1 gathers only grasslands populations, whereas group 2 includes populations from agricultural grounds. As expected, *Vt* shows larger sizes of all floral elements (e.g., CO_LE 22.02 ± 2.24 mm, CO_WI 17.18 ± 2.07 mm, SE_LE 13.57 ± 1.85 mm) and peduncle (79.09 ± 24.17 mm), and a greater number of half lamina teeth (6 ± 1). As for the stigmatic chamber entrance and the stylar flap, *Vt* differs from the other two species (Table S2a). The indices per population provide helpful information on the corolla and spur shape. In *Va*, the ratio corolla length/width is higher (from 1.29 ± 0.11 to 1.43 ± 0.20) than in *Vk* (from 1.14 ± 0.12 to 1.23 ± 0.10) except for VA-ST, VA-LA, and VA-SCI, having a corolla similar in size and shape (ratio ranging from 1.10 ± 0.11 to 1.23 ± 0.21) to *Vk*. VA-SG shows the highest ratio spur length/width (3.10 ± 0.29), which is greater than in VT-PG (2.84 ± 0.46), denoting a relatively thin spur (Table S2c).

**Figure 1:**
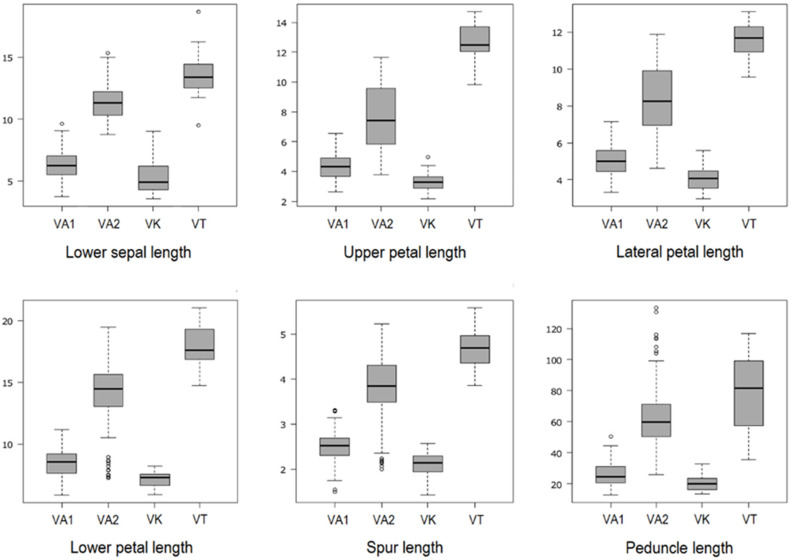
Box plots of the six main lengths. VA1: *V. arvensis* from grasslands, VA2: *V. arvensis* from fields, VK: *V. kitaibeliana*, VT: *V. tricolor*. All measurements are in mm. Whiskers indicate the 10–90 percentile, outliers are plotted as individual circles. Statistical significance with *p* < 0.0001, as determined by ANOVA (see also Table 1).

Afterward, we gathered the populations, by species, in the following groups: group VA1, the five *Va* populations with smaller flowers, group VA2, all the remaining populations of *Va*, group VK, all populations of *Vk*, and group VT, the *Vt* single population. Table 1 summarises the mean values of floral (Table 1a) and vegetative (Table 1b) features per group (those with a star are plotted in Figure 1). Values of indices are not displayed. Tukey’s test shows statistically significant differences (*p*-value < 0.05) among almost all the quantitative floral mean values. All values in VA1 are lower than in VA2. VK has the significantly smallest flowers (Tukey’s test *p* < 0.05) (Figure 1). VA1 shows significant differences in mean petals width from VA2 and VT, not VK. VA2 significantly differs from VT in all the floral features (Tukey’s test *p* < 0.05), including the petals mean width (Table 1a).

Table 1b shows statistically significant differences (Tukey’s test *p*-value < 0.05) in peduncle length, which reaches the maximum value in VT (79.09 ± 24.17 mm) and the minimum value in VK (20.15 ± 4.44 mm) (Figure 1), and in the half lamina teeth number, with the maximum value in VT (5 ± 1) and the minimum value in VK (1 ± 1). The mean number of stipule external lobes shows no significant differences between VA2 and VT. The significantly different mean values of leaf and stipule size between VA1 (LA_LE 6.52 ± 2.79 mm, LA_WI 3.71 ± 1.28 mm, PET_LE 4.70 ± 2.20 mm) and VA2 (LA_LE 21.67 ± 6.77 mm, LA_WI 10.02 ± 4.23 mm, PET_LE 9.56 ± 4.15 mm) confirm the clear split-up shown by the small-flowered *V. arvensis* from grasslands compared to the much-developed *V. arvensis* from fields and fallow lands. Leaves size and stipules mean length (9.61 ± 3.93 mm) in VA1 do not significantly differ from those in VK (LA_LE 5.57 ± 2.59 mm, LA_WI 2.99 ± 0.85 mm, PET_LE 4.01 ± 1.47 mm and ST_LE 7.46 ± 2.95 mm). VT shows significantly larger average leaf sizes (LA_LE 28.44 ± 5.10 mm, LA_WI 12.43 ± 2.58 mm, and PET_LE 11.04 ± 3.62 mm). The only leaves in VA/B1 show overall higher mean values (29.06 ± 7.37 mm, 13.23 ± 6.13 mm, and 10.81 ± 6.54 mm, respectively) than those of VT. No significant differences emerge among groups considering the indices’ values (means not shown), except for corolla length/width, lateral petal length/width, lower petal length/width, and spur length/width.

### 2.2. Multivariate Analysis

**Figure 2:**
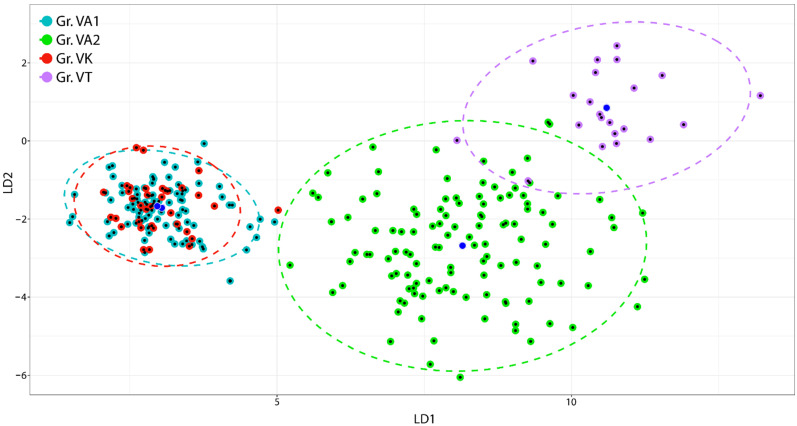
Scatterplot of first and second linear discriminant projections of floral variables, four groups and three species. Dots depict individual samples; group centroids are in blue, ellipses fitted at the 95% confidence level. VA1 and VA2: *V. arvensis*, VK: *V. kitaibeliana*, VT: *V. tricolor*.

We obtained the best two LDA applied separately to floral and vegetative variables, considering four different groups of samples: group VA1 combining individuals of all the *Va* populations growing on grasslands, group VA2 comprising all remaining *Va* individuals, group VK comprising all *Vk* individuals, and group VT with the *Vt* unique population. For all conducted LDA were computed the confusion matrices. The Supplementary Material (Table S3a) provides the confusion matrices for the best two LDA. The first and second discriminant directions account for about 86% and 9.7% of the trace’s total proportion, respectively. The first discriminant projection (LD1) separates groups VA1 and VK (as a whole) from groups VA2 and VT (Figure 2).

Almost all floral variables are significantly and positively correlated with the first discriminant projections (Table 2a): the corresponding correlations are greater than 0.7 but the correlation with spur width (0.36). All the considered floral variables are discriminating factors of the four groups; lower sepal length (0.96) and lower petal length (0.92) have the largest share in the discrimination. The second discriminant projection (LD2) separates group VT from the remaining ones (Figure 2). The only variables which are significantly correlated with the second discriminant projection are upper petal length (0.38), upper petal width (0.32), lateral petal length (0.21), lateral petal width (0.32), lateral petal upper half (0.26), and lower petal width (0.24).

**Figure 3:**
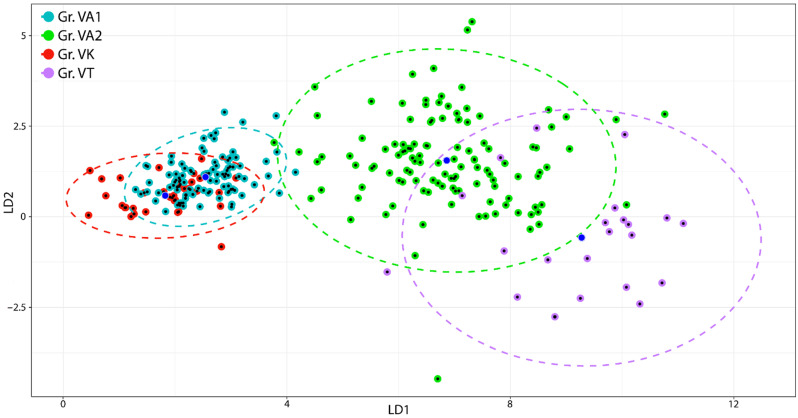
Scatterplot of first and second linear discriminant projections of vegetative variables, four groups and three species. Dots depict individual samples; group centroids are in blue, ellipses fitted at the 95% confidence level. VA1 and VA2: *V. arvensis*, VK: *V. kitaibeliana*, VT: *V. tricolor*.

We conducted the second LDA upon the vegetative variables. The sum of the proportion of trace of the first two linear discriminating axes is 97.73%. LD1 separates VA1 and VK (again, these two groups seem to belong to the same species) from group VA2 and group VT. LD2 separates groups VA1 and VK (as a unique group) and VA2 from group VT (Figure 3). Almost all variables are positively and significantly correlated with the first discriminating axis (the correlations are in general greater than 0.80) (Table 2b). Lamina length (0.92) and half lamina teeth number (0.91) have the largest share in the discrimination. The lowest, but still significant, correlations correspond to petiole length (0.68), stipule external lobes (0.54), and stipule internal lobes (0.45). All variables significantly contribute to discriminating the different groups along the first projection. When considering the second linear discriminating axis, only four variables have a significant correlation index: stipules external lobes (0.38), stipules length (0.36), half lamina teeth number (0.13), and petiole length (0.13). Overall, by analysing the position of the objects within the ellipses, no grouping of individuals by population is highlighted (Figure 2 and Figure 3).

**Figure 4:**
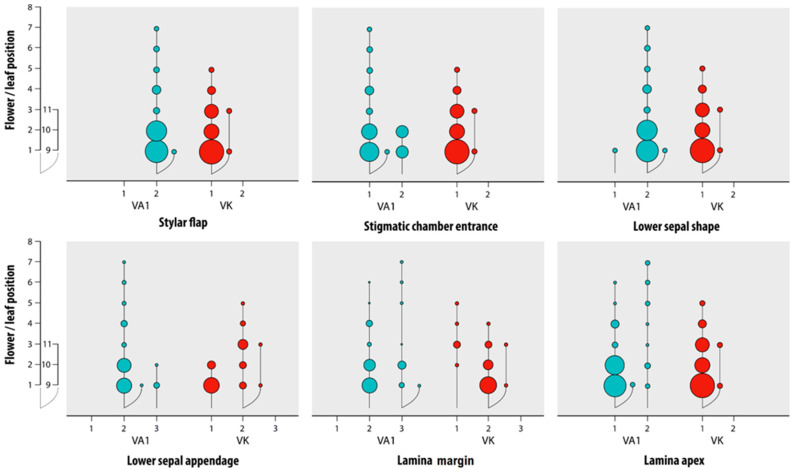
Graphical representation of the joint relative frequencies of the six most discriminating categorical variables in grassland-grown *V. arvensis* (VA1) and *V. kitaibeliana* (VK). Bubble plots show the joint relative frequencies of the features’ outcomes (x-axes) and the flower/leaf positions (y-axes) along the main stem (1–8) and the lateral branches (9–11). Bubbles of increasing size represent increasing frequency. Features’ outcomes: Stylar flap: 1 absent, 2 small and scarcely protruding; Stigmatic chamber entrance: 1 in front, 2 lightly oblique; Lower sepal shape: 1 ovate-lanceolate, 2 narrowly lanceolate-acuminate; Lower sepal appendage: 1 entire, 2 irregularly sinuate, 3 coarsely dentate; Lamina margin: 1 entire, 2 crenate, 3 dentate; Lamina apex: 1 rounded, 2 acute.

LDA did not discriminate groups VA1 and VK. The separation between these groups was ascertained using joint counts of categorical variables and considering each item’s position along the stem. Two-way summary tables of this analysis are reported in the Supplementary Material (Table S3b). Figure 4 shows the graphical representations of the most informative joint counts. In the pair flower/leaf position and stylar flap, the behaviour of this variable is different in VA1 and VK: feature 1 (absent) is absent in VA1, while in VK is absent the feature 2 (small and scarcely protruding). The pair flower/leaf position and stigmatic chamber entrance show the absence of feature 2 (lightly oblique) in VK. Feature 1 (ovate-lanceolate) of the variable lower sepal shape is almost absent in VA1 (it is only present with a very low frequency of 0.02, in correspondence with feature 1 of flower/leaf position), on the contrary, feature 2 (narrowly lanceolate-acuminate) is always absent in VK. Feature 1 (entire) of the lower sepal appendage is absent in VA1, while feature 3 (irregularly sinuate) is absent in VK. The behaviour of the variable lamina margin is different in VA1 and VK: feature 1 (entire) is absent in VA1, feature 3 (dentate) is absent in VK for all features of the variable flower/leaf positions. Finally, feature 2 (acute) of the variable lamina apex is absent for all VK flower/leaf positions.

**Figure 5:**
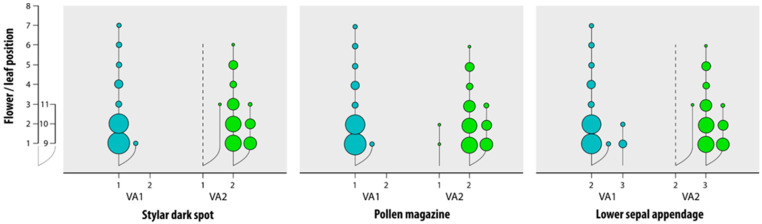
Graphical representation of the joint relative frequencies of the three most discriminating categorical variables in grassland-grown (VA1) and field-grown (VA2) *V. arvensis*. Bubble plots show the joint relative frequencies of the features’ outcomes (x-axes) and the flower/leaf positions (y-axes) along the main stem (1–8) and the lateral branches (9–11). Bubbles of increasing size represent increasing frequency. Features’ outcomes: Stylar dark spot: 1 absent, 2 present; Pollen magazine: 1 open, 2 closed; Lower sepal appendage: 1 entire, 2 irregularly sinuate, 3 coarsely dentate.

To confirm the discrimination between groups VA1 and VA2, the analysis of the relative joint frequencies for categorical variables was also made. For the stylar dark spot and pollen magazine, feature 1 (absent and open, respectively) is almost absent in VA2 while feature 2 (present and closed, respectively) is absent in VA1. As for the lower sepal appendage, feature 3 (coarsely dentate) is absent in VA1, while it is present in VA2, and feature 2 (irregularly sinuate) is absent in VA2 while it is present in VA1; finally, feature 1 (entire) is absent from both groups (Figure 5).

The Spearman’s rho coefficient was computed for the pair’s flower/leaf position and those significant discriminating variables found with LDA. Although the values are not very high, positive and negative associations also emerge (Table 3).

Data refer only to the combinations of flowers/leaves on the main axis. On lateral branches, there were suitable numbers only for a few populations of group VA1.

Concerning floral characters, in field-grown *Va* (VA2), there are no significant correlations. Positive significant correlation (*p*-value < 0.0001) is instead in *Vk* (lower sepal length, ρs 0.62; upper petal length, ρs 0.57; lateral petal upper half, ρs 0.65), while in grassland-grown *Va* (VA1) values are significant (*p*-value < 0.0001) but lower (ρs > 0.35). In *Vt*, a significant negative correlation emerges for corolla length (ρs −0.70) and lower petal length (ρs −0.66). Among vegetative characters, the highest positive association is in *Vk* for lamina length (ρs 0.75, *p*-value < 0.0001) and stipule length (ρs 0.68, *p*-value < 0.0001). The highest negative association is in *Vt*, for peduncle length (ρs −0.84, *p*-value < 0.0001) and stipule length (ρs −0.68, *p*-value < 0.0001).

## 3. Discussion

### 3.1. Reliability of the Morphological Characters

*V. kitaibeliana* and *V. arvensis* are widely recognized in European floras and checklists, in all the main international directories and online databases. Recent Floras little focused on their discriminating characters. Among them, Flora d’Italia [17], endorsing the account of Flora Europea [18], pointed out the sepal length (6–12 mm in *Va* vs. 3–6 mm in *Vk*) and the lamina length and apex (lamina > 1 cm, acute in *Va* compared to <1 cm, rounded in *Vk*). The New Flora of the British Isles [55] reported an 8–20 mm almost flat corolla and a 2–4 mm spur in *Va* vs. a 4–8 mm concave corolla and a 1–2 mm spur in *Vk* (currently known as *V. nana* (DC.) Le Jolis [26]). Yousefi et al. [24] distinguished *Va* by a 2.0–3.0 cm stipule with a leaf-like divided middle segment and a leaf margin crenulate, compared to *Vk* having a 0.5–1.0 cm stipule with a lanceolate middle segment, larger than laterals, and a leaf margin entire. Flora Iberica [43] first distinguished the wider flowers from the smallest (based on the flower position on the stems) in providing both *Va* and *Vk* measurements. Among them, in the larger flowers, the length of the lower petal (5–12 mm in *Va* vs. 1–5 mm or larger in *Vk*), or the length and shape of the lower sepal (7–15.5 mm with a 1–3 toothed appendix in *Va* vs. 5–12 mm with a rounded appendage or with only one slightly marked tooth in *Vk*).

In *Vk*, the current measurements and literature nearly overlapped; data confirm that the dwarf pansy is morphologically uniform [19]. It is not the case in *Va*, where measurements were higher than the literature in our field samples and lower in grassland samples. Such low values of the flowers and leaves size in *Va* were reported herein for the first time. The new records help quantify the “high” variability intra- and inter- population of *Va*, whose range of variation seems to include almost entirely the one of *Vk*.

Both LDA, in fact, mainly arranged samples according to an increasing dimensional gradient of flowers and leaves without discriminating between *Vk* and the smaller individual plants of *Va*. It means that quantitative characters had a more similar trend in individual plants of the two species inhabiting similar habitats than in plants of the same species from different habitats (cf. Figure 1). As a practical implication in identification, we cannot rely on these characters to decide whether a single or few grassland-grown individuals belong to the dwarf or the field pansy. More generally, quantitative characters reported as crucial for species delimitation in annual pansies (i.e., corolla size, spur length, leaf and stipule length, and peduncle length) proved to be not entirely informative to distinguish *Va* from *Vk* individual plants.

Qualitative characters allowed a more refined analysis (Figure 4 and Figure 5). Features of reproductive structures proved crucial for discriminating pansies [5,7], as already pointed out by authors from very early classifications quoted therein. Instead, previous literature did not consider the sepals appendage shape a suitable diagnostic feature in *V. arvensis* s.l. [15,56] as shown by our results.

Based on Spearman’s correlation coefficients (Table 3), we did not observe any general pattern of variation in flowers and leaves size along the main stem, but only patterns within species, particularly in *Vk* (bearing stems with maximum five flowers) and in grassland-grown *Va* (maximum of seven flowers/leaves), secondly in *Vt* (maximum of eight flowers). In *Vk* (secondly in grassland-grown *Va*), a significant positive correlation emerged between flower position and lower sepal, upper and lateral petal, and lateral petal upper half indicating a gradual increase in the size of subsequent flowers. Results in *Vk* could be distorted by the relatively limited number of positions examined, given the reduced growth of most plants in the wild. In *Vt*, we confirm the increasing reduction from lower to upper positions of the corolla size, lower petal length, and peduncle length [7,14,57]. The lateral petal lower half (the petal “nail”) did not vary with the flower position in the studied sample. In contrast, the petal upper half was positively correlated with flower position and petal length itself (see Table S1). In fact, in pansies, the tuft of papillose hairs that delimits the petal nail is closely related to the position and size of androecium and gynoecium in each species that are conservative features [31,57,58]. The spur length had no diagnostic value in Va: in the mean values, it showed a wide range that overlaps those in *Vk* and *Vt* (Figure 1).

As leaves and stipules develop their typical shape after a given vegetative period [7,14], the current assessment of the leaves underlying the flower peduncles could be reliable. In *Vk* (secondly in grassland-grown *Va*), leaves and stipules progressively lengthen, and the number of stipule lobes increased (especially in grassland-grown *Va*). In field-grown *Va*, as in *Vt*, the leaves become progressively narrower, reducing the petiole and stipules length from lower to upper positions; this variation is influenced by the gradual change (seasonal induced) of the micro-environmental conditions and the increased plant size (spatial induced) [57,59]. Indeed, leaf size and shape varied within all the studied samples (Table S2). Thus, data confirm that leaf length is of little value in delimiting taxa [31]. The stipule shape is considered an essential character in the *Viola* species [7,14,17], although hard to evaluate in the smallest leaves of *Vk* and *Va*. In the three species, it ranges from pinnatifid, prevailing in the smaller leaves, to digitate, especially in the larger ones. Accordingly, the stipule shape cannot be considered a species-specific character; instead, it seems related to the leaf size. Results do not entirely agree with Erben’s statements and our previous study [7]. As highlighted [60], when more material is examined, characters previously used to separate taxa may no longer be suitable; any differences could emerge only after a more comprehensive study.

We found values of standard floral indices (ratios petals/sepals, petals length/width, and spur length/width) similar in the pairs that are grassland-grown *Va*/*Vk* and field-grown *Va*/*Vt*, not adding any information for identification purposes. They are regarded statistically significant among species of the section *Melanium*, including *Viola aetolica* Boiss. and Heldr. and *Viola elegantula* Schott [9]. Some principal Floras also consider some indices as useful [43,55,61,62]. Thus, their reliability in identifying pansies remains somewhat questionable.

**Figure 6:**
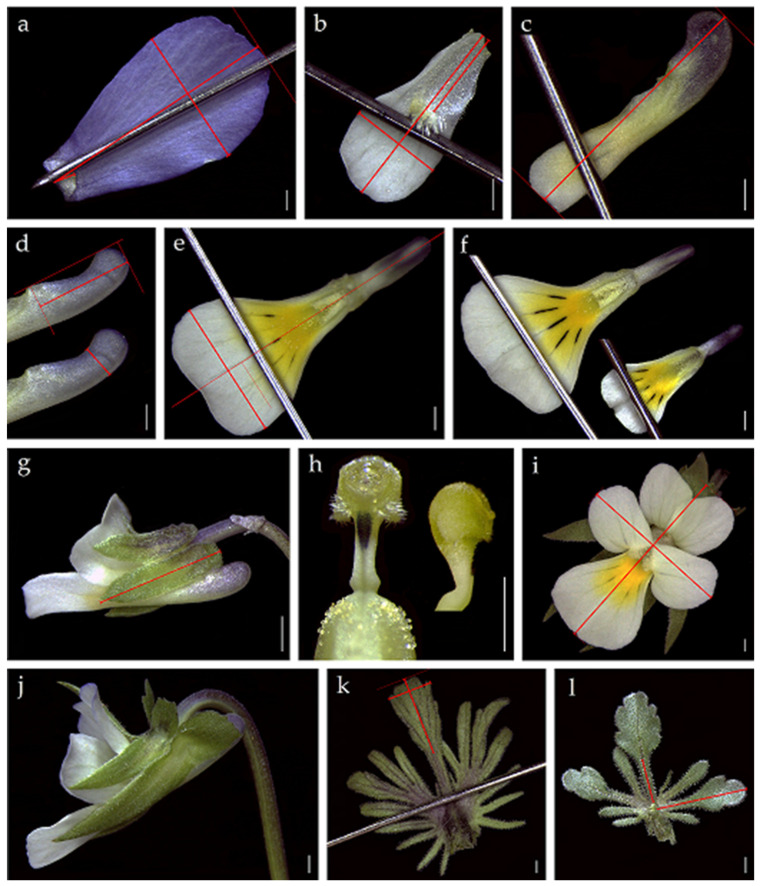
The main morphological features from fresh material (scale bar = 1 mm): (**a**) upper petal length and width; (**b**) lateral petal length, width and lower half; (**c**) lower petal length; (**d**) lower petal width; (**e**) spur length and width; (**f**) pollen magazines (open and closed); (**g**) lower sepal length; (**h**) stylar dark spot (present); stylar flap (small and scarcely protruding); (**i**) corolla length and width; (**j**) lower sepal shape (narrowly lanceolate-acuminate), and appendage (irregularly sinuate); (**k**) lamina length and width; stipule shape (pinnately lobed); stipule external lobes number; (**l**) half lamina teeth number, petiole length, stipule length, stipule midlobe margin (crenate-dentate), stipule shape (palmately lobed). Needles, required to highlight details in the smallest elements, are evident in some pictures.

As far as we know, no statistical evidence exists on the variation of the bracts position on the peduncle in the annual pansies. Some authors pointed out that it could be an exploitable feature to distinguish *Va* from *Vk* [35,54]. We noticed that the bracts position varied from the bend just below the flower in *Vk* (Figure 6g) to far below the bend of peduncle in *Va* (bracts not visible in Figure 6j) and *Vt*. In the last two species, the distance increased distinctly in the fruiting peduncle, while in *Vk* it did not. It remains unclear whether the bracts’ position is related to the length of flowering/fruiting peduncles or not.

Table 4 summarises the essential qualitative features of *Vk* and *Va*. Accordingly, the grasslands’ small-sized and small-flowered *Va* individuals differ from the fields *Va* individuals by the lower sepal appendage never coarsely dentate, and the stylar dark spot absent. They differ from *Vk* by the lower sepal narrowly lanceolate-acuminate instead of ovate-lanceolate, the stylar flap small and scarcely protruding instead of absent, and the lamina margin crenate to dentate, never entire. Overall, a set of robust characters that would reliably discriminate all the *Va* individuals from *Vk* have not emerged from the study and observing flowering plants at natural sites. On the contrary, the mean values of the measured characters per species significantly differ (Table 1). The latter results are consistent with previous reports (cf. [7,14]).

### 3.2. Explaining Variations in V. arvensis and Distinction of an Eco-Phenotype

According to the literature and the current survey, the most frequent morphotype of *Va* in the Italian territory is the weedy type from agricultural habitats (the group VA2 in the analysis). This type, to which keys refer, is the most commonly found in herbaria.

*Va* samples from agricultural habitats showed phenotypic variation even across populations located at very reduced distances (cf. sub-populations VA-B/1 and VA-B/2). Fields are seen, in fact, as spatially and temporally heterogeneous environments [47,63].

The study highlighted the presence of a double pollination strategy in *Va* rather than a predominantly autogamous strategy, as reported by [10,30,40]. The coexistence of the features “absent” in the stylar dark spot, “open” in pollen magazine, and “in front” in stigmatic chamber entrance in most of the flowers in the weak grassland-grown *Va* samples suggested an autogamous pollination strategy. This strategy would not seem to prevail in plants from agricultural habitats, where the corresponding features “present”, “closed”, and “lightly oblique” far prevail, suggesting a heterogamous strategy (Figure 5, Table S2b).

In agricultural habitats, we found both populations, with corolla as long as or shorter than calyx (e.g., VA-SG and VA-AZ) and corolla rather exceeding calyx (e.g., VA-BS and VA-B/1). We do not assign populations with larger corolla to *V. arvensis* subsp. megalantha Nauenb. (2*n* = 34) given its distribution range, flowers shape, and habit (isotypus, Switzerland, Bern-Wabern, südl. Ortsausgang, 1984, J.D. Nauenburg, GOET, scan seen) (acronym according to [64]). The current presence of this pansy in Italy is questionable [44,65]. It is probably linked to a few areas in the colline–montane belt in central Europe [60,66]. The lectotype of *V. arvensis* (Basel, inter segetes, C. Bauhin, BAS, scan seen) shows flowers with the corolla fully included in the calyx. However, J.D.Nauenburg [36] suggested the total phenotypic variation of *Va* was already known to C. Bauhin as his original sample also includes individual plants with large corolla. The intermediate between *Va* and *Vt* of hybrid origin (*V. x contempta* Auct., 2*n* = 30) is not yet ascertained in Italy [15,21,31], unlike Pignatti’s statement [17]. A cytological approach could only verify it since its real presence. In northern Lazio, pansies with corolla exceeding calyx (length 15–20 mm) inhabiting fields on travertine layers are 2*n* = 34 [67]. In our opinion, the field-grown *Va* from Central Italy, having the corolla exceeding calyx, could currently be seen as a possible introgressant to *Vt* rather than a different taxon.

Populations inhabiting dry-grasslands and other near-natural grassy open places (the group VA1 in the analysis), even relatively close to previous field-grown populations (e.g., VA-SCI vs. VA-SG), were adapted to such a different habitat. Plants were small, mainly self-pollinating, and with earlier bloom, showing strong convergence with *Vk*.

The features found in these grassland-grown populations support the differentiation of an undescribed eco-phenotype within *Va* resulting from adaptation to dry grassy fallows and open grasslands on carbonate soil. This pansy participates in the earliest stages of the therofitic succession in patches of loose soil and calcareous debris, among tufts of the competing grasses (i.e., *Bromus* spp., *Koeleria* spp., *Phleum* spp., *Poa* spp.), covered or not by moss layers. In Central Italy, it grows along a wide altitudinal amplitude ranging from 45 to 1.180 m of elevation (Table 5).

We previously suggested its presumed hybrid origin [7], which is indicated generically in Flora d’Italia [17]. However, there has never been cytological evidence of natural interspecific hybrids between *Va* and *Vk* in plant material from Central Italy. Such a hybrid has been suspected in Turkey [32]. In Britain, *Va* hybridises readily with *Vt* [31], but the record of hybrids with *Vk* was never confirmed, although plants having habit and stipules of *Va* and flowers and fruits of *Vk* have been reported in the 1950s [35].

Our study suggested that the intraspecific variation in *Va* could result, to some extent, from genetic divergence caused by selection pressures cf. [47]. To date, there is no molecular genetic evidence for this assumption due to the scarcity of appropriate genetic markers and the poor availability of extensive datasets. However, our studies in progress are very promising, underlining the usefulness (even the need) of joined morphological and molecular approaches in species delimitation. Defining the actual distribution range of this neglected pansy and uncovering an eventual genetic basis explaining this variability would be crucial to assume the presence of an original wild race and a weedy race within *Va* (T. Marcussen in litt.). A similar event does already occur in *V. rafinesquei* [20].

Little is known so far on the distribution of this type in Italy and Europe, as it has been misidentified for a long time. As far as we know, at least one specimen in ZAGR (No. 43887, Velebit Mountains chain, 2016, S. Bogdanovic and M. Rat, sub *V. kitaibeliana*) would confirm this morphotype in Croatia, but it certainly grows elsewhere. The current findings could be congruent with other European records of *Vk* or *Va* from dry slopes and shallow soil on rocks (e.g., [24,68,69,70]). Notably, in Flora Helvetica [61], lower petals of about 10 mm, as long as the lower sepals in *Vk*, may indicate possible confusion with *Va*.

*Vk* was recently assigned to the risk category of “endangered” on the Italian regional scale because of the continuing decline of the estimated area of occupancy (AOO) and the low number of locations in its geographic range [71,72]. Indeed, the Italian AOO of *Vk* is overestimated due to the proven species misidentification (cf. images in [65], except the specimen from CAT, and revised specimina visa in [7]). *Vk* can only be confirmed in a few Italian locations (cf. our further revisions in APP, AO, CAT, PI, RO, UTV).

In Central Italy, grassland-grown *Va* is more successful than *Vk*, taking advantage of man’s activities. *Va* produces more abundant and larger seeds and has a wide distribution within its range [10,21,35]. Notably, in the Apennines stony pastures where human intrusions have altered habitats, the *Va* increasing occurrence is one of the causes of the *Vk* negative trend. Thus, the conservation of *Vk* appears determined mainly by the nature of the uses of lands. The current study provides a baseline for planning a broader survey in the field and herbaria to evaluate whether the Italian increasingly reduced occurrence of the dwarf pansy constitutes a general trend. If confirmed, at least the southern European range of *Vk* will suffer further severe reductions. To preserve this species and optimize local conservation efforts, a better definition of its European area of occupancy would be needed.

## 4. Materials and Methods

### 4.1. Sampling Sites and Field Collection

We checked fourteen wild populations from Central Italy, i.e., from Lazio and Tuscany regions, mainly on limestone and travertine layers (Table 5), possibly with known cytotype, representing the greatest variability of the species highlighted in the primary literature. The adopted taxonomic circumscription followed [17]. Species identification was performed using [14,17], and our previous outcomes [7,10]. The plant materials were compared with the type specimens in the herbaria M, BAS, and LINN; additional information was taken from specimens in UTV.

Plants were monitored in the wild throughout the flowering period (from February to May) during 2018–2019. Ten to twenty living plants per population, depending on the population size, were randomly selected. One to two fully bloomed flowers with the correspondent leaves per plant were harvested from the stem (or the main lateral branches) to account for their variability, avoiding damaged organs. The species/plants were labelled, and the flower/leaf positions along the stem were numbered. Overall, 272 flowers and as many leaves were used in the analyses, divided as follows: 10 wild populations of *Va*, 15–20 individual plants per population, a total of 215 flowers/leaves, and three populations of *Vk*, 10–15 individual plants per population, 35 flowers/leaves. In addition, a *Vt* single population from the Tyrrhenian Antiapennine sector was included as reference material for comparison (20 individual plants, 22 flowers/leaves). Specimens were preserved as dried vouchers in UTV for later checks. Most populations were karyologically known [10,69]. We did not deem cytological analysis necessary to confirm agricultural ground’s VA-BS, VA-SG, and VA-B/1-2 populations., Recent comparative palynology and seed morphology have confirmed their belonging to *V. arvensis* [10].

### 4.2. Morphometry and Numerical Analyses

#### 4.2.1. Characters Scored for Classical Morphometric Analyses

We use the term variable for any categorical (binary or multistate) or quantitative (continuous, discrete) attribute. Floral variables were examined on fresh samples under a Leica M60 stereomicroscope at 6.4× to 50× magnification. High-definition microphotographs were obtained using a Leica IC80HD digital camera (e.g., Figure 6), and measurements were performed on the images using the application LAS ver. 3.8. for Leica Instruments. Measurements of the whole corolla, sepal, and peduncle (after gently straightening it) were gathered on fresh material using a digital calliper with 0.01 mm precision. Measurements of leaves underlying peduncles were performed on material removed from fresh plants, then pressed and dried. Data were gathered for 21 quantitative and 11 categorical variables previously considered as systematically crucial [7,14,16,53,54,62,73]. (Figure 6, Table 6). According to the literature, ten ratios were derived (Table 6), presuming to reflect among-species differences better than the individual variables [9]. The floral and vegetative datasets were treated separately. Neither the fruiting peduncle length nor the bracts position on the peduncle was individually computed. The latter, more reliable when computed in the fruit-bearing peduncle than in the floral [7,54], was unavailable given the adopted sampling protocol.

#### 4.2.2. Statistical Analyses

All computations have been carried out in R [74]. We firstly analysed floral and vegetative variables using univariate and bivariate statistical techniques. Pearson’s correlation index was preliminarily performed for quantitative variables (original values) without distinction of species or populations to verify, if there are, their linear relationships. Some descriptive statistics (arithmetic mean, minimum-maximum, standard deviation) were computed per population, per species (*Vk* and *Vt*), and per groups within species (two groups in *Va*). We also performed the Analysis of Variance (ANOVA), which used the F-test to verify the equality of the means of the different groups (once the assumptions of normality of data and homoscedasticity have been checked using Pearson’s kurtosis index and Barlett’s test, respectively). We carried out Tukey’s test to assess groups statistically different. Boxplots were used to visually examine the variation of the main continuous variables among groups. We used multivariate statistical techniques to investigate both quantitative and nominal observations, following a multistep approach. First, we used Linear Discriminant Analysis (LDA) to analyse quantitative variables and uncover the group structure [75]. We used bivariate joint counts and Spearman’s rank correlation coefficient (ρs) to investigate categorical variables.

Discriminant analysis assigns a new observation to the most appropriate group using the features of the groups themselves [75]; LDA performs this task by data projections that best separate the groups. The separation between groups is assessed with the ratio of the variance between the projected group means and the variance of the projection. The higher this ratio, the better the separation. The R function lda{MASS} [76] was used to compute the best discriminating projections. We first conducted LDA over 11 floral variables: SE_LE, UPP_LE, UPP_WI, LAP_LE, LAP_WI, LAP_UPHA, LAP_LOHA, LOP_LE, LOP_WI, SP_LE, and SP_WI (see Table 6 for acronyms explanation). We conducted the second LDA upon eight vegetative variables: PED_LE, LA_LE, LA_WI, PET_LE, HALA_TE, ST_LE, ST_EXLO, ST_INLO. Corolla values have been removed from LDA to avoid data duplication. Indices did not add information and have been removed as well. Finally, the statistical significance of the correlations between the projected data and the original variables was computed with Fisher’s transform [77]. We used joint relative frequencies of all categorical variables to assess the distinction between *Va* and *Vk*. Bubble plots were used as a graphical representation of the most discriminant relative joint counts. Larger bubbles indicate higher joint relative frequencies, while the presence or the absence of bubbles denotes the presence or the absence of the corresponding features’ outcomes. We used formal hypothesis testing to assess the strength and the direction of the relationship, if any, between flower/leaf position and other floral/vegetative variables, computing the Spearman’s rho, a measure of monotonic association [78]. The Spearman’s rho is the correlation between the variables’ ranks [79]. We tested the nullity of Spearman’s rho of flower/leaf position and other floral and vegetative variables using the algorithm AS 89 with an Edgeworth series approximation as used by the R command cor.test{stats}.

## 5. Conclusions

The study, based on extended field-work and a large mass of characters explored statistically, aimed at evaluating the similarity or the hidden diversity in *V. arvensis* and *V. kitaibeliana*, the two possibly more problematic species within the ***V. t****ricolor* species complex. We attempted to verify whether morphological variation could be higher within *Va* than between *Va* and *Vk* and find new and more useful species-delimitating characters. Data were collected from wild populations in Central Italy. Results fit into the generally accepted picture of low resolution in the distinctness among species of the section ***Melanium*** and led us to reconsider the worth of certain characters in delimiting related pansies. Indeed, multivariate analysis of a large set of morphological variables could not satisfactorily distinguish *Va* from *Vk*. None of the quantitative characters, single or in combination, can be used as diagnostic to differentiate completely single *Va* individuals from *Vk* and vice versa. On the other hand, quantitative characters showed significant differences in the mean values per population at a specific and intraspecific level. There are, instead, species–specific differences in the sepal shape (we assessed the left lower sepal), in the lamina margin and apex (we set leaves underlying peduncles), and in some reproductive features. In fresh flowering plants, *Va* can be distinguished with certainty from *Vk* by the lower sepals shape ovate-lanceolate and the presence of the stylar flap, albeit scarcely protruding. Leaf margins and sepals shape are essential in ascribing the dried specimens to one or the other species.

An additional distinguishing character emerged from field observations to be statistically assessed: the position of the bracts in the fruit-bearing peduncles. Concerning *Vk*, given the threatened status in Italy and the limited resources in the wild, they are difficult to evaluate on adequate sample size.

This research contributes substantially to understanding intra- and inter- specific diversity patterns in such controversial material. Results showed that intraspecific variation of *Va* could be more significant than previously thought and higher than the interspecific variation between *Va* and *Vk*. Accordingly, a relatively identical flower and leaf morphology does not necessarily indicate species identity in the *V. tricolor* species complex. This fact possibly explains the ambiguous or erroneous references in literature and herbaria.

Results support the autonomy of an undescribed eco-phenotype in *Va*, linked to dry grasslands and pastures. It is reasonable to assume that in Italy, this type progressively replaces *Vk*, which appears less competitive in perturbed environments and richer soils. We do not exclude the occurrence in the Apennines of putative introgressive wild forms of *Va* into *Vk* we have not yet ascertained. Geographical boundaries of this wild *Va* type remain poorly known due to difficulties in taxonomic identification. Further material from Italian territories and European countries would therefore be studied. An accurate karyological and genetic characterization of a larger dataset could effectively complete the study and assign this pansy as an eventual wild race in addition to the well-known weedy race of *V. arvensis*.

It is worth underlining that the importance of this study is related to a general increased knowledge of the genus models of evolution and, more in general, to an assessment of regional biodiversity trajectories.

## Figures and Tables

**Table 1:** Comparison of quantitative floral (a) and vegetative (b) characters among the four studied groups of populations ^1^. All measurements are in mm (mean ± SD, with minimum-maximum values in brackets). Means with different superscript letters (^a–d^) within each variable significantly differ (Tukey’s test, *p*-value < 0.05). ANOVA results: *p*-value: probability; R2: fraction of the overall variance attributable to differences among the group means.

(a)											
Code	SE_LE *	UPP_LE *	UPP_WI	LAP_LE *	LAP_WI	LAP_UPHA	LAP_LOHA	LOP_LE *	LOP_WI	SP_LE *	SP_WI
**VA1**	6.31 ± 1.18 ^a^ (3.73–9.60)	4.34 ± 0.79 ^a^ (2.63–6.55)	2.35 ± 0.48 ^a^ (1.33–3.97)	5.01 ± 0.77 ^a^ (3.32–7.15)	2.48 ± 0.42 ^a^ (1.67–3.55)	2.67 ± 0.49 ^a^ (1.70–4.10)	2.33 ± 0.38 ^a^ (1.47–3.05)	8.48 ± 1.07 ^a^ (5.89–11.18)	3.45 ± 0.51 ^a^ (2.43–4.87)	2.51 ± 0.35 ^a^ (1.50–3.31)	1.53 ± 0.22 ^a^ (0.93–1.97)
**VA2**	11.31 ± 1.40 ^b^ (8.74–15.34)	7.61 ± 2.12 ^b^ (3.79–11.64)	4.93 ± 1.79 ^b^ (2.17–8.70)	8.29 ± 1.82 ^b^ (4.63–11.87)	4.38 ± 1.37 ^b^ (2.00–6.93)	5.36 ± 1.54 ^b^ (2.74–8.45)	2.93 ± 0.40 ^b^ (1.80–3.75)	13.95 ± 2.61 ^b^ (7.26–19.47)	7.04 ± 2.18 ^b^ (2.78–11.66)	3.79 ± 0.70 ^b^ (2.00–5.23)	1.65 ± 0.35 ^b^ (0.86–2.29)
**VK**	5.30 ± 1.34 ^c^ (3.57–9.03)	3.35 ± 0.63 ^c^ (2.18–4.98)	2.00 ± 0.39 ^a^ (1.44–2.85)	4.03 ± 0.61 ^c^ (2.98–5.57)	2.17 ± 0.38 ^a^ (1.54–3.27)	2.13 ± 0.35 ^c^ (1.56–3.17)	1.90 ± 0.32 ^c^ (1.29–2.69)	7.16 ± 0.58 ^c^ (5.90–8.23)	3.10 ± 0.35 ^a^ (2.44–3.78)	2.11 ± 0.26 ^c^ (1.43–2.57)	1.14 ± 0.19 ^c^ (0.67–1.48)
**VT**	13.57 ± 1.85 ^d^ (9.49–18.67)	12.56 ± 1.31 ^d^ (9.81–14.70)	8.30 ± 1.43 ^c^ (6.28–11.11)	11.55 ± 1.04 ^d^ (9.55–13.09)	6.87 ± 1.07 ^c^ (5.20–8.60)	8.36 ± 0.90 ^d^ (6.46–9.56)	3.19 ± 0.32 ^d^ (2.45–3.91)	17.87 ± 1.58 ^d^ (14.71–21.02)	10.76 ± 1.32 ^c^ (8.41–12.98)	4.70 ± 0.47 ^d^ (3.86–5.58)	1.70 ± 0.33 ^d^ (1.21–2.46)
**R^2^**	0.82	0.73	0.67	0.73	0.65	0.73	0.53	0.76	0.70	0.70	0.76
***p*** **-value**	<0.0001	<0.0001	<0.0001	<0.0001	<0.0001	<0.0001	<0.0001	<0.0001	<0.0001	<0.0001	<0.0001
**(b)**											
**Code**	PED_LE *	**LA_LE**	**LA_WI**		**PET_LE**	**ST_LE**	**HALA_TE**	**ST_EXLO**		**ST_INLO**	
**VA1**	26.60 ± 7.15 ^a^ (12.91- 50.40)	6.52 ± 2.79 ^a^ (2.65–15.65)	3.71 ± 1.28 ^a^ (1.80–7.82)		4.70 ± 2.20 ^a^ (1.52–13.65)	9.61 ± 3.93 ^a^ (3.88–24.91)	2 ± 1 ^a^ (1–4)	4 ± 1 ^a^ (2–8)		2 ± 1 ^a^ (1–3)	
**VA2**	63.80 ± 20.65 ^b^ (25.92–133.25)	21.67 ± 6.77 ^b^ (9.88–49.42)	10.02 ± 4.23 ^b^ (3.95–25.76)		9.56 ± 4.15 ^b^ (2.60–22.04)	23.89 ± 7.36 ^b^ (7.84–54.50)	4 ± 1 ^b^ (2–7)	5 ± 1 ^b^ (3–9)		2 ± 1 ^a^ (0–4)	
**VK**	20.15 ± 4.44 ^c^ (13.60–32.78)	5.57 ± 2.59 ^a^ (2.62–12.21)	2.99 ± 0.85 ^a^ (1.62–5.14)		4.01 ± 1.47 ^a^ (2.13–7.54)	7.46 ± 2.95 ^a^ (3.82–14.88)	1 ± 1 ^c^ (0–3)	3 ± 1 ^c^ (2–5)		2 ± 1 ^a^ (1–3)	
**VT**	79.09 ± 24.17 ^d^ (35.59–116.85)	28.44 ± 5.10 ^c^ (18.08–36.43)	12.43 ± 2.58 ^c^ (8.37–18.76)		11.04 ± 3.62 ^b^ (4.25–17.70)	24.23 ± 5.24 ^b^ (13.33–34.78)	5 ± 1 ^d^ (4–7)	5 ± 1 ^b^ (3–8)		2 ± 1 ^a^ (1–3)	
**R^2^**	0.64	0.73	0.57		0.40	0.63	0.72	0.29		0.24	
***p*** **-value**	<0.0001	<0.0001	<0.0001		<0.0001	<0.0001	<0.0001	<0.0001		<0.0001	

^1^ See Figure 1 and Section 4.2.1 for acronyms explanation * Variables plotted in Figure 1.

**Table 2:** Statistical significance of the correlations between the projected data and the original floral (a) and vegetative (b) variables where LD1 and LD2 are the first and the second directions, respectively ^1^ (*** *p*-value < 0.001, * *p*-value < 0.05, n.s. not significant).

	LD1			LD2		
Variable	Cor	Fisher Transf	Signif	Cor	Fisher Transf	Signif
**(a)**						
SE_LE	0.96	30.96	***	−0.03	−0.48	n.s.
UPP_LE	0.86	21.08	***	0.38	6.48	***
UPP_WI	0.83	19.73	***	0.32	5.45	***
LAP_LE	0.89	23.22	***	0.21	3.42	***
LAP_WI	0.82	19.07	***	0.32	5.37	***
LAP_UPHA	0.88	22.93	***	0.26	4.40	***
LAP_LOHA	0.74	15.42	***	−0.05	−0.85	n.s.
LOP_LE	0.92	25.79	***	0.10	1.63	n.s.
LOP_WI	0.87	21.96	***	0.24	4.01	***
SP_LE	0.88	22.34	***	0.09	1.42	n.s.
SP_WI	0.36	6.26	***	−0.07	−1.07	n.s.
**(b)**						
PED_LE	0.86	21.08	***	0.10	1.57	n.s.
LA_LE	0.92	26.00	***	0.04	0.70	n.s.
LA_WI	0.81	18.71	***	0.09	1.53	n.s.
PET_LE	0.68	13.61	***	0.13	2.06	*
ST_LE	0.83	19.58	***	0.36	6.14	***
HALA_TE	0.91	24.94	***	0.13	2.15	*
ST_INLO	0.45	8.04	***	0.07	1.18	n.s.
ST_EXLO	0.54	9.95	***	0.38	6.52	***

^1^ See Section 4.2.1 for acronyms explanation.

**Table 3:** Spearman’s correlation coefficients (ρs) for pairs of variables, flower position/other floral (a) and vegetative (b) variables, in the four groups ^1^. Significance: *** *p*-value < 0.001, ** *p*-value < 0.01, * *p*-value < 0.05, n.s. not significant.

	VA1		VA2		VK		VT	
Variables	ρs	Signif	ρs	Signif	ρs	Signif	ρs	Signif
**(a)**								
SE_LE	0.46	***	0.05	n.s.	0.62	***	−0.17	n.s.
UPP_LE	0.31	**	0.00	n.s.	0.59	***	−0.47	*
UPP_WI	0.40	***	−0.04	n.s.	0.38	*	−0.48	*
LAP_LE	0.36	***	−0.05	n.s.	0.50	**	−0.40	n.s.
LAP_WI	0.40	***	−0.04	n.s.	0.44	**	0.66	**
LAP_UPHA	0.36	***	−0.05	n.s.	0.65	***	−0.47	*
LAP_LOHA	0.25	*	−0.09	n.s.	0.27	n.s.	0.01	n.s.
LOP_LE	0.22	*	−0.03	n.s.	0.10	n.s.	−0.66	***
LOP_WI	0.25	*	0.02	n.s.	0.00	n.s.	−0.51	*
SP_LE	0.21	*	−0.01	n.s.	0.01	n.s.	0.02	n.s.
CO_LE	0.29	**	0.01	n.s.	0.36	*	−0.70	***
CO_WI	0.24	*	0.05	n.s.	0.44	**	−0.64	**
**(b)**								
PED_LE	0.18	n.s.	−0.31	**	0.23	n.s.	−0.84	***
LA_LE	0.36	***	0.02	n.s.	0.75	***	0.13	n.s.
LA_WI	−0.29	**	−0.47	***	−0.18	n.s.	−0.58	**
PET_LE	0.10	n.s.	−0.53	***	0.29	n.s.	−0.54	**
ST_LE	0.27	**	−0.23	*	0.68	***	−0.68	***
HALA_TE	−0.38	***	−0.35	***	−0.26	n.s.	−0.45	*
ST_INLO	0.58	***	−0.09	n.s.	0.18	n.s.	0.12	n.s.
ST_EXLO	0.52	***	−0.33	***	0.54	**	−0.52	*

^1^ The variable FL-LE_PO (flower/leaf position) and the variables significantly correlated with LD1 in both LDA, applied to floral (a) and vegetative (b) characters, were considered, with the addition of CO_LE and CO_WI. See Section 4.2.1 for acronyms explanation.

**Table 4:** Qualitative features of the *V. arvensis* morphotypes compared to *V. kitaibeliana*, useful for quick visual recognition of taxa.

Characters	Grassland-Grown *V. arvensis*	Field and Fallow Land-Grown *V. arvensis*	*Viola kitaibeliana*
Lower sepal	narrowly lanceolate-acuminate	narrowly lanceolate-acuminate	ovate-lanceolate
Lower sepal appendage	irregularly sinuate, rarely coarsely dentate	coarsely dentate	entire to irregularly sinuate
Pollen magazine	open	almost always closed	open
Stylar flap	small and scarcely protruding	almost always small and scarcely protruding	absent
Stylar dark spot	absent	present	absent
Lamina margin	crenate to dentate	crenate to dentate	entire to crenate
Lamina apex	almost always rounded	acute to rounded	apex rounded
Bracts in the fruit-bearing peduncle	below bend ^1^	far below bend ^1^	just below fruit or at the bend ^1^

^1^ To be confirmed with statistical data.

**Table 5:** The studied populations sorted per species with their ID ^1^.

ID	Sampling Site	Habitat	Elevation	Coordinates	Voucher Code
*Viola arvensis* subsp. *arvensis* (*Va*)					
**VA-ST**	Ex Polverificio Stacchini, Tivoli Terme (Roma)	Open stony grassland on travertine	45	41°56′29″ N, 12°43′24″ E	UTV 37188
**VA-LA**	Zona Laghi, Tivoli Terme (Roma)	Shrubby grassland on travertine	66	41°57′44″ N, 12°43′11″ E	UTV 37190
**VA-SCI**	Loc. Le Sparagine (SCI), Tivoli Terme (Roma)	Waste ground on travertine	66	41°57′46″ N, 12°42′50″ E	UTV 37859
**VA-BS**	Bassano Scalo, Orte (Viterbo)	Olive grove	73	42°27′50″ N, 12°21′51″ E	UTV 36771
**VA-SG**	San Gregorio da Sassola (Roma)	Resting field	143	41°54′02″ N, 12°48′45″ E	UTV 38286
**VA-AZ**	Azienda Agr. Riello, Viterbo (Viterbo)	Resting field	303	42°25′35″ N, 12°04′50″ E	UTV 30441
**VA-B/1**	Loc. Bagnaccio, Viterbo (Viterbo)	Cereal field	320	42°28′59″ N, 12°03′52″ E	UTV 36783
**VA-B/2**	Loc. Bagnaccio, Viterbo (Viterbo)	Resting field	319	42°27′42″ N, 12°03′54″ E	UTV 36780
**VA-CE**	Monte Cetona, Sarteano (Siena)	Stony grassland on calcareous soil	1100	42°55′52″ N, 11°52′32″ E	UTV 30445
**VA-TA**	Monte Tancia, Monte San Giovanni in Sabina (Rieti)	Stony grassland on calcareous soil	1180	42°19′04″ N, 12°44′41″ E	UTV 32125
***Viola kitaibeliana*** ** subsp. *kitaibeliana* (*Vk*)**					
**VK-BS**	Bassano Scalo, Orte (Viterbo)	Arid and stony grassland on calcareous soil	70	42°29′12″ N, 12°19′28″ E	UTV 30152
**VK-NE**	Loc. Cerreta, Nespolo (Rieti)	Open grassland in rural environment on calcareous soil	1040	42°09′46″ N, 13°04′48″ E	UTV 29512
**VK-NA**	Monte Navegna, Varco Sabino (Rieti)	Stony grassland on calcareous soil	1184	42°14′03″ N, 12°59′34″ E	UTV 32126
***Viola tricolor*** ** subsp.* tricolor* (*Vt*)**					
**VT-PG**	Poggio Nibbio, Viterbo (Viterbo)	Grassy edge in semi-natural land on volcanic soil	885	42°21′42″ N, 12°10′17″ E	UTV 36752

^1^ Elevation is given as meters above sea level, geographic coordinates (latitude and longitude) are in DMS (WGS-84). Voucher code is given for at least one specimen per population.

**Table 6:** Floral and vegetative morphological variables and indices (with acronyms and states) considered in the analyses ^1^.

Quantitative Variables				
**Continuous (mm)**				
CO_LE	Corolla length *	SP_LE	Spur length	
CO_WI	Corolla width *	SP_WI	Spur width	
LAP_LE	Lateral petal length	UPP_LE	Upper petal length	
LAP_LOHA	Lateral petal lower half	UPP_WI	Upper petal width	
LAP_UPHA	Lateral petal upper half	LA_LE	Lamina length	
LAP_WI	Lateral petal width	LA_WI	Lamina width	
LOP_LE	Lower petal length	PED_LE	Peduncle length	
LOP_WI	Lower petal width	PET_LE	Petiole length	
SE_LE	Lower sepal length	ST_LE	Stipule length	
**Discrete**				
HALA_TE	Half lamina teeth number	ST_INLO	Stipule internal lobes number	
ST_EXLO	Stipule external lobes number			
**Categorical variables**				
PO_MA	Pollen magazine: 1 open, 2 closed			
SE_AP	Lower sepal appendage: 1 entire, 2 irregularly sinuate, 3 coarsely dentate			
SE_SH	Lower sepal shape:1 ovate-lanceolate, 2 narrowly lanceolate-acuminate			
ST_CH	Stigmatic chamber entrance (front view):1 in front, 2 lightly oblique (intermediate), 3 upward			
ST_DS	Stylar dark spot: 1 absent, 2 present			
STY	Stylar flap:1 absent, 2 small and scarcely protruding, 3 conspicuous			
LA_AP	Lamina apex: 1 rounded, 2 acute			
LA_ED	Lamina margin: 1 entire, 2 crenate, 3 dentate			
ST_ED	Stipule midlobe margin: 1 entire, 2 crenate-dentate			
ST_SH	Stipule shape: 1 palmately lobed, 2 pinnately lobed			
FL-LE_PO	Flower/leaf position: on main stems 1 to 8, on lateral branchs 9 to11			
**Indices**				
CO_LE/WI	Corolla length/width	LOHA/UPHA	Lateral petal lower half/upper half	
LAP_LE/WI	Lateral petal length/width	UPP_LE/WI	Upper petal length/width	
LOP/SE_LE	Lower petal/lower sepal length	LA/PET_LE	Lamina/petiole length	
LOP_LE/WI	Lower petal length/width	LA_LE/WI	Lamina length/width	
SP_LE/WI	Spur length/width	PED/LE_LE	Peduncle/leaf length	

^1^ Variables not used in multivariate analyses are marked with a star. See also Figure 6.

## Data Availability

The original data set used in this study is available on request from the corresponding author.

## Supplementary Materials

The following are available online at https://www.mdpi.com/article/10.3390/plants11030379/s1, Table S1: Pearson’s correlation between pairwise combinations of 21 quantitative variables: most floral variables and several vegetative variables were highly and positively correlated (r > 0.80) to each other, no noticeable correlations were detected by considering floral and vegetative variables in pairs; Table S2: The morphological features among the 14 investigated populations sorted per species, a. quantitative characters in mm, b. Qualitative characters (except FL-LE_PO, not evaluable), c. indices. Tables are analysed in the Results. Table S3: This file holds two sections: (1) Confusion matrices for the two best linear discriminant analysis upon the floral variables (Table S3_1a) and the vegetative variables (Table S3_1b) respectively; (2) Two-way summary tables of the most informative joint counts.
