# Supplementary material for: Classical Morphometrics in V. arvensis and V. kitaibeliana (Viola sect. Melanium) Reveals Intraspecific Variation with Implications for Species Delimitation: Inferences from a Case Study in Central Italy
Source: Plants (Basel). 2022 Jan 29;11(3):379. doi: 10.3390/plants11030379 (PMC8838537; doi:10.3390/plants11030379)
Supplement: Supplementary file 1 [file plants-11-00379-s001.zip › Table S1.pdf]

Supplementary Material Table S1 - Pearson's correlation between pairwise combinations of 21 quantitative variables. Boldfaced values indicate significant correlation (r > 0,80). See Table 2 in the text for abbreviations.

	**SE_LE**	**UPP_LE**	**UPP_WI**	**LAP_LE**	**LAP_WI**	**LAP_UPHA**	**LAP_LOHA**	**LOP_LE**	**LOP_WI**	**SP_LE**	**SP_WI**	**CO_LE**	**CO_WI**
**SE_LE**	**1.000000**												
**UPP_LE**	0.795219	**1.000000**											
**UPP_WI**	0.767889	**0.971618**	**1.000000**										
**LAP_LE**	**0.824890**	**0.981928**	**0.963332**	**1.000000**									
**LAP_WI**	0.756892	**0.969443**	**0.985303**	**0.966967**	**1.000000**								
**LAP_UPHA**	**0.808676**	**0.986994**	**0.971336**	**0.990561**	**0.971895**	**1.000000**							
**LAP_LOHA**	0.731746	0.776492	0.750060	**0.847335**	0.764862	0.766548	**1.000000**						
**LOP_LE**	**0.852464**	**0.938389**	**0.924728**	**0.966415**	**0.923821**	**0.947655**	**0.856209**	**1.000000**					
**LOP_WI**	0.782906	**0.966352**	**0.975605**	**0.972463**	**0.976917**	**0.974987**	0.778695	**0.954565**	**1.000000**				
**SP_LE**	**0.823939**	**0.857832**	**0.845770**	**0.889261**	**0.838314**	**0.860505**	**0.832223**	**0.949765**	**0.869099**	**1.000000**			
**SP_WI**	0.632454	0.708327	0.702917	0.761128	0.717932	0.705097	**0.834188**	**0.825797**	0.730204	**0.848489**	**1.000000**		
**CO_LE**	**0.844230**	**0.959987**	**0.944325**	**0.950410**	**0.933111**	**0.961841**	0.726518	**0.927521**	**0.946516**	**0.842071**	0.640834	**1.000000**	
**CO_WI**	**0.813392**	**0.959402**	**0.951032**	**0.941752**	**0.946074**	**0.957153**	0.704026	**0.903538**	**0.943662**	**0.819357**	0.634373	**0.973676**	**1.000000**
**PED_LE**	**0.809610**	0.757793	0.733517	0.786303	0.737861	0.779297	0.665004	0.799392	0.750765	0.753211	0.592770	0.787876	0.765058
**LA_LE**	**0.910518**	0.762379	0.719208	0.784992	0.707114	0.783478	0.642385	0.799437	0.735926	0.751662	0.547085	**0.801943**	0.766992
**LA_WI**	0.764290	0.648915	0.616444	0.690227	0.623496	0.677416	0.609626	0.712215	0.629883	0.661198	0.562662	0.684955	0.646976
**PET_LE**	0.694826	0.584801	0.566393	0.612616	0.561263	0.592638	0.574667	0.625178	0.548203	0.582215	0.540178	0.620034	0.590957
**HALA_TE**	0.781922	0.669602	0.645968	0.697077	0.634077	0.683963	0.616466	0.721653	0.657512	0.672482	0.521212	0.725607	0.688909
**ST_LE**	**0.864035**	0.687312	0.659466	0.731144	0.652498	0.713099	0.662953	0.757931	0.668814	0.718621	0.595542	0.726028	0.690383
**ST_INLO**	0.459071	0.283779	0.295344	0.306936	0.262087	0.295081	0.295648	0.305423	0.260307	0.278777	0.160939	0.340522	0.325402
**ST_EXLO**	0.604879	0.465555	0.473884	0.510904	0.466558	0.480800	0.531179	0.522942	0.448998	0.505780	0.488584	0.459309	0.440938

	**PED_LE**	**LA_LE**	**LA_WI**	**PET_LE**	**HALA_TE**	**ST_LE**	**ST_INLO**	**ST_EXLO**
**PED_LE**	**1.000000**							
**LA_LE**	**0.815623**	**1.000000**						
**LA_WI**	**0.805422**	**0.836973**	**1.000000**					
**PET_LE**	0.734056	0.724427	**0.851118**	**1.000000**				
**HALA_TE**	0.715876	0.735742	0.784201	0.697990	**1.000000**			
**ST_LE**	**0.823382**	**0.914693**	**0.881803**	**0.859507**	0.752632	**1.000000**		
**ST_INLO**	0.325332	0.461816	0.417505	0.432877	0.464210	0.464862	**1.000000**	
**ST_EXLO**	0.536655	0.581793	0.610399	0.625615	0.568729	0.692398	0.589871	**1.000000**
