# Supplementary material for: Classical Morphometrics in V. arvensis and V. kitaibeliana (Viola sect. Melanium) Reveals Intraspecific Variation with Implications for Species Delimitation: Inferences from a Case Study in Central Italy
Source: Plants (Basel). 2022 Jan 29;11(3):379. doi: 10.3390/plants11030379 (PMC8838537; doi:10.3390/plants11030379)
Supplement: Supplementary file 1 [file plants-11-00379-s001.zip › Table S2.pdf]

Supplementary Material Table S2 – The morphological features among the 14 investigated populations sorted per species. Abbreviation for populations as in Table 1 in the text.

SM S2a - Quantitative characters in mm (as mean ± SD, with minimum-maximum values in brackets)

	Continuous characters																		Discrete characters		
	Corolla length	Corolla width	Upper petal length	Upper petal width	Lateral petal length	Lateral petal width	Lateral petal upper half	Lateral petal lower half	Lower petal length	Lower petal width	Spur length	Spur width	Lower sepal length	Lamina length	Lamina width	Peduncle length	Petiole length	Stipule length	Half lamina teeth number	Stipule external lobes number	Stipule internal lobes number
Code	CO_LE	CO_WI	UPP_LE	UPP_WI	LAP_LE	LAP_WI	LAP_UPHA	LAP_LOHA	LOP_LE	LOP_WI	SP_LE	SP_WI	SE_LE	LA_LE	LA_WI	PED_LE	PET_LE	ST_LE	HALA_TE	ST_EXLO	ST_INLO
VA-ST	5.91 ± 1.15<br>(3.61-7.81)	4.93 ± 0.82<br>(3.69-6.75)	4.00 ± 0.73<br>(3.04-5.29)	2.26 ± 0.46<br>(1.56-3.28)	4.83 ± 0.82<br>(3.79-6.48)	2.48 ± 0.46<br>(1.88-3.55)	2.53 ± 0.47<br>(1.90-3.45)	2.30 ± 0.39<br>(1.82-3.04)	8.24 ± 1.02<br>(7.20-10.48)	3.34 ± 0.55<br>(2.71-4.60)	2.56 ± 0.26<br>(2.21-3.14)	1.55 ± 0.21<br>(1.20-1.91)	5.68 ± 1.32<br>(3.73-8.19)	6.17 ± 2.28<br>(2.74-11.81)	3.83 ± 1.47<br>(1.80-7.41)	26.37 ± 5.66<br>(19.11-37.40)	4.91 ± 2.34<br>(2.10-10.63)	8.98 ± 3.64<br>(3.88-16.23)	2 ± 1<br>(1-3)	4 ± 1<br>(3-7)	1 ± 1<br>(1-2)
VA-LA	6.64 ± 1.09<br>(5.20-9.02)	5.43 ± 0.73<br>(4.64-6.96)	4.92 ± 0.59<br>(3.63-5.85)	2.59 ± 0.39<br>(1.94-3.24)	5.55 ± 0.54<br>(4.23-6.47)	2.64 ± 0.34<br>(2.15-3.25)	3.12 ± 0.41<br>(2.25-3.89)	2.42 ± 0.24<br>(1.98-2.95)	8.90 ± 0.74<br>(7.60-10.76)	3.66 ± 0.42<br>(2.76-4.36)	2.60 ± 0.21<br>(2.28-3.28)	1.61 ± 0.15<br>(1.40-1.95)	6.84 ± 1.11<br>(5.63-8.61)	8.69 ± 3.85<br>(4.03-15.65)	2.91 ± 0.76<br>(1.96-4.77)	32.90 ± 8.44<br>(18.22-50.40)	4.35 ± 1.92<br>(2.37-8.60)	10.91 ± 3.88<br>(6.28-19.07)	2 ± 1<br>(1-2)	5 ± 1<br>(2-5)	2 ± 0<br>(1-2)
VA-SCI	6.07 ± 1.61<br>(3.24-8.99)	5.47 ± 1.10<br>(3.62-7.10)	4.63 ± 0.89<br>(3.44-6.55)	2.59 ± 0.63<br>(1.72-3.97)	5.23 ± 0.88<br>(4.05-7.15)	2.59 ± 0.50<br>(2.00-3.45)	2.88 ± 0.56<br>(1.97-4.10)	2.35 ± 0.42<br>(1.49-3.05)	8.57 ± 1.29<br>(6.47-11.18)	3.49 ± 0.51<br>(2.76-4.59)	2.49 ± 0.50<br>(1.54-3.31)	1.57 ± 0.26<br>(1.13-1.97)	6.51 ± 1.63<br>(4.35-9.60)	6.11 ± 2.81<br>(2.65-11.44)	4.01 ± 1.19<br>(2.90-7.00)	25.71 ± 7.51<br>(13.90-41.03)	4.58 ± 2.35<br>(1.52-9.69)	9.70 ± 4.67<br>(4.21-17.79)	3 ± 1<br>(2-4)	4 ± 1<br>(3-8)	2 ± 1<br>(1-3)
VA-TA	7.16 ± 1.18<br>(4.58-9.24)	5.52 ± 0.68<br>(4.05-6.76)	4.01 ± 0.78<br>(2.63-5.55)	2.13 ± 0.44<br>(1.33-2.94)	4.67 ± 0.76<br>(3.32-6.16)	2.31 ± 0.41<br>(1.67-3.2)	2.45 ± 0.39<br>(1.70-3.17)	2.21 ± 0.44<br>(1.47-2.99)	8.13 ± 1.23<br>(5.89-10.56)	3.35 ± 0.60<br>(2.43-4.87)	2.42 ± 0.38<br>(1.50-3.02)	1.46 ± 0.24<br>(0.93-1.89)	6.04 ± 0.58<br>(4.79-7.13)	5.75 ± 1.67<br>(3.45-9.72)	3.41 ± 1.08<br>(1.89-5.68)	24.31 ± 4.75<br>(18.44-39.48)	4.28 ± 1.58<br>(2.25-9.17)	8.58 ± 2.40<br>(4.65-14.57)	3 ± 1<br>(1-4)	4 ± 1<br>(2-5)	2 ± 1<br>(1-2)
VA-CE	6.40 ± 0.90<br>(5.05-8.94)	4.81 ± 0.74<br>(3.84-6.09)	4.33 ± 0.60<br>(3.45-5.87)	2.29 ± 0.32<br>(1.78-2.80)	4.96 ± 0.52<br>(4.04-5.80)	2.46 ± 0.32<br>(1.87-3.01)	2.53 ± 0.29<br>(2.06-3.04)	2.43 ± 0.33<br>(1.81-3.02)	8.73 ± 0.80<br>(7.18-9.87)	3.46 ± 0.39<br>(2.76-4.21)	2.50 ± 0.34<br>(1.89-3.04)	1.52 ± 0.22<br>(1.01-1.78)	6.62 ± 1.04<br>(4.60-8.41)	6.17 ± 2.47<br>(3.52-13.21)	4.51 ± 1.36<br>(2.93-7.82)	24.67 ± 6.74<br>(12.91-37.40)	5.52 ± 2.82<br>(2.79-13.65)	10.29 ± 5.06<br>(5.57-24.91)	3 ± 1<br>(2-4)	4 ± 1<br>(2-5)	1 ± 1<br>(1-2)
VA-B/1	15.13 ± 3.31<br>(10.27-21.52)	10.95 ± 2.31<br>(7.37-15.72)	8.47 ± 2.01<br>(4.97-11.39)	5.34 ± 1.44<br>(2.75-7.73)	9.16 ± 1.42<br>(6.24-11.17)	4.85 ± 1.10<br>(2.99-6.57)	6.13 ± 1.44<br>(3.51-8.32)	3.03 ± 0.31<br>(2.41-3.53)	15.28 ± 1.55<br>(12.27-17.64)	7.89 ± 1.80<br>(5.31-11.66)	4.23 ± 0.39<br>(3.50-4.81)	1.71 ± 0.26<br>(1.16-2.06)	12.24±1.17<br>(10.49-15.34)	29.06 ± 7.37<br>(16.67-49.42)	13.23 ± 6.13<br>(5.02-25.76)	97.46 ±19.29<br>(62.94-133.25)	10.81 ± 6.54<br>(2.60-22.04)	29.79 ± 10.46<br>(15.57-54.50)	4 ± 1<br>(2-6)	5 ± 1<br>(4-8)	2 ± 1<br>(0-4)
VA-B/2	16.34 ± 2.18<br>(11.65-19.35)	12.37 ± 2.36<br>(7.52-15.57)	9.29 ± 1.18<br>(6.25-10.93)	6.78 ± 1.14<br>(4.41-8.7)	9.77 ± 0.95<br>(7.63-11.54)	5.75 ± 0.72<br>(4.32-6.93)	6.57 ± 0.82<br>(4.73-8.10)	3.20 ± 0.24<br>(2.69-3.75)	15.69 ± 1.35<br>(13.11-17.92)	8.98 ± 1.17<br>(6.50-11.46)	4.15 ± 0.43<br>(3.38-5.03)	1.99 ± 0.15<br>(1.65-2.25)	10.89 ± 1.33<br>(8.96-13.25)	17.39 ± 5.38<br>(9.88-31.33)	8.93 ± 2.12<br>(5.83-15.35)	61.71 ±16.69<br>(25.92-96.63)	10.49 ± 3.82<br>(3.54-18.26)	23.15 ± 6.46<br>(7.84-34.32)	5 ± 1<br>(3-6)	6 ± 1<br>(3-9)	2 ± 1<br>(1-4)
VA-BS	14.12 ± 3.35<br>(9.24-21.52)	10.21 ± 3.31<br>(6.47-15.02)	7.89 ± 2.14<br>(5.32-11.64)	4.94 ± 1.52<br>(3.20-7.98)	8.63 ± 1.73<br>(6.58-11.87)	4.34 ± 1.39<br>(2.83-6.61)	5.63 ± 1.60<br>(3.72-8.45)	3.01 ± 0.29<br>(2.45-3.69)	14.23 ± 1.27<br>(12.53-16.99)	7.37 ± 1.98<br>(5.07-11.25)	3.61 ± 0.28<br>(3.08-3.95)	1.69 ± 0.13<br>(1.40-2.07)	10.69 ± 0.96<br>(9.64-12.89)	23.49 ± 3.70<br>(16.69-31.83)	10.98 ± 4.04<br>(5.61-21.96)	57.89 ± 6.79<br>(50.45-72.83)	9.32 ± 3.36<br>(5.47-18.25)	23.74 ± 4.29<br>(16.77-33.64)	5 ± 1<br>(4-6)	6 ± 1<br>(4-7)	3 ± 0<br>(2-3)
VA-AZ	10.39 ± 1.70<br>(7.75-14.51)	7.68 ± 1.30<br>(5.69-10.89)	5.24 ± 0.82<br>(3.79-7.05)	2.96 ± 0.58<br>(4.47-2.17)	6.14 ± 1.06<br>(4.63-8.14)	2.96 ± 0.60<br>(2.00-4.49)	3.61 ± 0.66<br>(2.74-5.07)	2.53 ± 0.48<br>(1.80-3.29)	10.64 ± 2.70<br>(7.26-14.89)	4.42 ± 1.20<br>(2.78-6.76)	2.95 ± 0.69<br>(2.00-4.19)	1.42 ± 0.43<br>(0.86-2.29)	11.12 ± 1.28<br>(8.74-14.37)	19.98 ± 6.28<br>(11.70-38.37)	10.50 ± 3.19<br>(5.33-16.93)	51.81 ±12.02<br>(26.69-81.08)	9.41 ± 3.27<br>(3.92-16.26)	23.15 ± 6.81<br>(12.89-39.87)	5 ± 1<br>(3-7)	6 ± 1<br>(3-8)	3 ± 1<br>(2-4)
VA-SG	13.84 ± 2.63<br>(10.25-19.74)	10.23 ± 2.12<br>(7.02-14.93)	7.28 ± 1.46<br>(5.04-10.95)	4.58 ± 1.33<br>(2.59-6.90)	7.84 ± 1.07<br>(5.98-10.06)	3.94 ± 0.91<br>(2.64-5.86)	4.95 ± 0.95<br>(3.66-7.16)	2.89 ± 0.25<br>(2.32-3.34)	14.31 ± 1.75<br>(11.19-19.47)	6.62 ± 1.16<br>(4.39-8.67)	4.19 ± 0.49<br>(3.23-5.23)	1.36 ± 0.19<br>(1.03-1.78)	11.97 ± 1.67<br>(8.92-14.99)	20.21 ± 4.71<br>(12.00-29.19)	6.44 ± 2.43<br>(3.95-15.33)	57.82 ±11.90<br>(43.39-88.92)	7.32 ± 2.75<br>(3.18-16.3)	19.66 ± 5.41<br>(12.60-32.98)	4 ± 1<br>(2-5)	4 ± 1<br>(3-6)	2 ± 1<br>(1-3)
VK-BS	4.94 ± 2.50<br>(3.54-9.83)	4.02 ± 2.10<br>(2.88-8.78)	3.02 ± 0.45<br>(2.18-3.96)	1.84 ± 0.30<br>(1.44-2.43)	3.71 ± 0.50<br>(2.98-4.90)	2.00 ± 0.34<br>(1.54-2.90)	1.93 ± 0.23<br>(1.56-2.27)	1.78 ± 0.32<br>(1.29-2.69)	7.07 ± 0.57<br>(6.37-8.04)	3.12 ± 0.39<br>(2.50-3.78)	2.08 ± 0.26<br>(1.63-2.49)	1.07 ± 0.21<br>(0.67-1.48)	4.65 ± 1.15<br>(3.81-9.03)	3.71 ± 0.65<br>(2.62-4.73)	3.10 ± 0.78<br>(1.96-4.81)	18.96 ± 3.61<br>(13.60-25.51)	3.68 ± 1.41<br>(2.39-7.54)	5.58 ± 1.40<br>(3.87-9.14)	2 ± 1<br>(1-3)	3 ± 1<br>(2-4)	2 ± 0<br>(1-3)
VK- NA	5.99 ± 0.54<br>(4.86-6.62)	5.22 ± 0.35<br>(4.43-5.66)	3.48 ± 0.32<br>(2.90-4.10)	2.21 ± 0.41<br>(1.73-2.85)	4.29 ± 0.41<br>(3.47-4.79)	2.35 ± 0.23<br>(2.01-2.62)	2.28 ± 0.23<br>(1.84-2.46)	2.01 ± 0.24<br>(1.63-2.34)	7.31 ± 0.60<br>(5.90-8.13)	2.98 ± 0.28<br>(2.44-3.40)	2.16 ± 0.31<br>(1.43-2.57)	1.24 ± 0.14<br>(0.97-1.41)	6.20 ± 1.05<br>(3.90-7.76)	7.67 ± 2.17<br>(4.40-12.21)	3.39 ± 0.74<br>(2.57-5.14)	24.57 ± 3.41<br>(20.81-32.78)	4.71 ± 1.54<br>(2.48-6.93)	10.07 ± 2.59<br>(6.15-14.88)	2 ± 0<br>(1-2)	4 ± 1<br>(3-5)	2 ± 1<br>(1-3)
VK-NE	6.29 ± 1.62<br>(4.13-8.42)	5.52 ± 1.20<br>(4.19-7.17)	4.18 ± 0.73<br>(2.80-4.98)	2.14 ± 0.45<br>(1.44-2.84)	4.61 ± 0.65<br>(3.59-5.57)	2.41 ± 0.49<br>(1.77-3.27)	2.53 ± 0.37<br>(2.00-3.17)	2.08 ± 0.35<br>(1.50-2.42)	7.21 ± 0.66<br>(6.51-8.23)	3.21 ± 0.29<br>(2.83-3.48)	2.12 ± 0.19<br>(1.96-2.45)	1.20 ± 0.10<br>(1.07-1.33)	5.90 ± 1.32<br>(3.57-7.15)	7.95 ± 2.66<br>(3.69-11.15)	2.00 ± 0.40<br>(1.62-2.72)	16.52 ± 2.43<br>(13.81-19.56)	3.92 ± 1.39<br>(2.13-5.94)	9.05 ± 3.14<br>(3.82-13.37)	0 ± 0<br>(0-0)	4 ± 1<br>(3-5)	2 ± 0<br>(2-2)
VT-PG	22.02 ± 2.24<br>(16.58-25.44)	17.18 ± 2.07<br>(12.97-20.48)	12.56 ± 1.31<br>(9.81-14.70)	8.30 ± 1.43<br>(6.28-11.11)	11.55 ± 1.04<br>(9.55-13.09)	6.87 ± 1.07<br>(5.2-8.60)	8.36 ± 0.90<br>(6.46-9.56)	3.19 ± 0.32<br>(2.45-3.91)	17.87 ± 1.58<br>(14.71-21.02)	10.76 ± 1.32<br>(8.41-12.98)	4.70 ± 0.47<br>(3.86-5.56)	1.70 ± 0.33<br>(1.21-2.46)	13.57 ± 1.85<br>(9.49-18.67)	28.44 ± 5.10<br>(18.08-36.43)	12.43 ± 2.58<br>(8.37-18.76)	79.09 ± 24.17<br>(35.59-116.85)	11.04 ± 3.62<br>(4.25-17.70)	24.23 ± 5.24<br>(13.33-34.78)	6 ± 1<br>(4-7)	5 ± 1<br>(3-8)	2 ± 1<br>(1-3)

SM S2b - Categorical characters (except FL-LE_PO, not evaluable)

	Pollen magazine	Lower sepal appendage	Lower sepal shape	Stigmatic chamber entrance (front view)	Stylar dark spot	Stylar flap size	Lamina apex	Lamina margin	Stipule midlobe margin	Stipule shape
Code	PO_MA	SE_AP	SE_SH	ST_CH	ST_DS	STY	LA_AP	LA_ED	ST_ED	ST_SH
VA-ST	Open<br>100%	Irregularly sinuate<br>100%	Narrowly lanceolate-acuminate<br>88.89%<br>Ovate-lanceolate<br>11.11%	In front<br>100%	Absent<br>100%	Small and scarcely protruding<br>100%	Rounded<br>100%	Crenate<br>100%	Entire<br>77.70%<br>Crenate-dentate<br>22.30%	Pinnatifid<br>88.89%<br>Digitate<br>11.11%
VA-LA	Open<br>100%	Irregularly sinuate<br>100%	Narrowly lanceolate-acuminate<br>100%	In front<br>100%	Absent<br>100%	Small and scarcely protruding<br>100%	Acute<br>55.50%<br>Rounded<br>44.50%	Dentate<br>100%	Entire<br>94.44%<br>Crenate-dentate<br>5.56%	Pinnatifid<br>100%
VA-SCI	Open<br>100%	Irregularly sinuate<br>100%	Narrowly lanceolate-acuminate<br>100%	In front<br>100%	Absent<br>100%	Small and scarcely protruding<br>100%	Rounded<br>93.30%<br>Acute<br>6.70%	Crenate<br>100%	Crenate-dentate<br>100%	Pinnatifid<br>66.67%<br>Digitate<br>33.33%
VA-TA	Open<br>100%	Irregularly sinuate<br>88.46%<br>coarsely dentate<br>11.54%	Narrowly lanceolate-acuminate<br>100%	Lightly oblique<br>100%	Absent<br>100%	Small and scarcely protruding<br>100%	Rounded<br>92.30%<br>Acute<br>7.70%	Crenate<br>76.90%<br>Dentate<br>23.10%	Crenate-dentate<br>82.60%<br>Entire<br>17.40%	Pinnatifid<br>100%
VA-CE	Open<br>100%	Irregularly sinuate<br>73.68%<br>coarsely dentate<br>26.32%	Narrowly lanceolate-acuminate<br>100%	In front<br>100%	Absent<br>100%	Small and scarcely protruding<br>100%	Rounded<br>100%	Crenate<br>95.00%<br>Dentate<br>5.00%	Crenate-dentate<br>89.40%<br>Entire<br>10.60%	Digitate<br>89,5%<br>Pinnatifid<br>10,5%
VA-B/1	Closed<br>100%	coarsely dentate<br>100%	Narrowly lanceolate-acuminate<br>100%	In front<br>50.00%<br>Lightly oblique<br>50.00%	Present<br>100%	Small and scarcely protruding<br>95.00%<br>Conspicuous<br>5.00%	Acute<br>60.00%<br>Rounded<br>40.00%	Dentate<br>50.00%<br>Crenate<br>50.00%	Crenate-dentate<br>80.00%<br>Entire<br>20.00%	Digitate<br>100%
VA-B/2	Closed<br>100%	coarsely dentate<br>100%	Narrowly lanceolate-acuminate<br>100%	Lightly oblique<br>71.43%<br>In front<br>28.57%	Present<br>100%	Small and scarcely protruding<br>92.86%<br>Conspicuous<br>7.14%	Rounded<br>60.70%<br>Acute<br>39.30%	Dentate<br>53,57%<br>Crenate<br>46.43%	Crenate-dentate<br>100%	Digitate<br>96.43%<br>Pinnatifid<br>3.57%
VA-BS	Closed<br>100%	coarsely dentate<br>100%	Narrowly lanceolate-acuminate<br>100%	In front<br>50%<br>Lightly oblique<br>50%	Present<br>100%	Small and scarcely protruding<br>95.84%<br>Conspicuous<br>4.16%	Rounded<br>66.60%<br>Acute<br>33.40%	Crenate<br>66.60%<br>Dentate<br>33,40%	Crenate-dentate<br>100%	Digitate<br>100%

VA-AZ	Closed<br>92.59%<br>Open<br>7.41%	coarsely dentate<br>100%	Narrowly lanceolate-acuminate<br>100%	In front<br>96.30%<br>Lightly oblique<br>3.70%	Present<br>96.35%<br>Absent<br>3.65%	Small and scarcely protruding<br>100%	Acute<br>100%	Dentate<br>100%	Crenate-dentate<br>92.59%<br>Entire<br>7.41%	Digitate<br>100%
VA-SG	Closed<br>100%	coarsely dentate<br>100%	Narrowly lanceolate-acuminate<br>100%	Lightly oblique<br>81.82%<br>In front<br>18.18%	Present<br>91.32%<br>Absent<br>8.68%	Small and scarcely protruding<br>95.46%<br>Conspicuous<br>4.54%	Acute<br>100%	Dentate<br>100%	Crenate-dentate<br>59.00%<br>Entire<br>41.00%	Digitate<br>90.91%<br>Pinnatifid<br>9.09%
VK-BS	Open<br>100%	Entire<br>84.21%<br>Irregularly sinuate<br>15.79%	Ovate-lanceolate<br>100%	In front<br>100%	Absent<br>100%	Absent<br>100%	Rounded<br>100%	Crenate<br>100%	Crenate-dentate<br>63.15%<br>Entire<br>36.85%	Digitate<br>100%
VK- NA	Open<br>100%	Irregularly sinuate<br>90.00%<br>Entire<br>10.00%	Ovate-lanceolate<br>100%	In front<br>100%	Absent<br>100%	Absent<br>100%	Rounded<br>100%	Crenate<br>100%	Entire<br>50.00%<br>Crenate-dentate<br>50.00%	Pinnatifid<br>100%
VK-NE	Open<br>100%	Irregularly sinuate<br>83.33%<br>Entire<br>16.67%	Ovate-lanceolate<br>100%	In front<br>100%	Absent<br>100%	Absent<br>100%	Rounded<br>100%	Entire<br>100%	Entire<br>100%	Pinnatifid<br>100%
VT-PG	Closed<br>100%	Irregularly sinuate<br>77.27%<br>coarsely dentate<br>22.73%	Narrowly lanceolate-acuminate<br>100%	Upward<br>100%	Present<br>100%	Conspicuous<br>100%	Acute<br>77.20%<br>Rounded<br>22.80%	Dentate<br>77.20%<br>Crenate<br>22.80%	Crenate-dentate<br>54.54%<br>Entire<br>45.46%	Digitate<br>77.27%<br>Pinnatifid<br>22.73%

SM S2c - Indices (as mean ± SD, with minimum-maximum values in brackets)

	Corolla length/width	Upper petal length/width	Lateral petal length/width	Lateral petal lower half/upper half	Lower petal length/width	Lower petal/lower sepal length	Spur length/width	Lamina length/width	Lamina/petiole length	Peduncle/leaf length
Code	CO_LE/WI	UPP_LE/WI	LAP_LE/WI	LOHA/UPHA	LOP_LE/WI	LOP/SE_LE	SP_LE/WI	LA_LE/WI	LA/PET_LE	PED/LE_LE
VA-ST	1.21 ± 0.19<br>(0.92-1.59)	1.78 ± 0.14<br>(1.49-2.00)	1.95 ± 0.10<br>(1.80-2.18)	0.92 ± 0.10<br>(0.75-1.09)	2.48 ± 0.13<br>(2.24-2.74)	1.49 ± 0.20<br>(1.16-1.93)	1.67 ± 0.16<br>(1.50-2.06)	1.72 ± 0.67<br>(1.05-2.93)	1.34 ± 0.32<br>(0.84-1.94)	2.67 ± 1.10<br>(1.50-6.32)
VA-LA	1.23 ± 0.21<br>(1.02-1.74)	1.91 ± 0.15<br>(1.68-2.19)	2.11 ± 0.19<br>(1.82-2.46)	0.79 ± 0.10<br>(0.64-1.02)	2.44 ± 0.15<br>(2.19-2.75)	1.32 ± 0.14<br>(1.07-1.53)	1.63 ± 0.15<br>(1.40-1.95)	3.08 ± 1.36<br>(1.55-5.64)	2.11 ± 0.88<br>(1.35-4.87)	2.94 ± 1.33<br>(1.06-5.68)
VA-SCI	1.10 ± 0.11<br>(0.90-1.28)	1.81 ± 0.16<br>(1.64-2.12)	2.03 ± 0.15<br>(1.75-2.21)	0.83 ± 0.16<br>(0.58-1.23)	2.46 ± 0.16<br>(2.15-2.73)	1.36 ± 0.24<br>(1.03-1.79)	1.59 ± 0.16<br>(1.34-1.93)	1.50 ± 0.47<br>(0.91-2.36)	1.40 ± 0.27<br>(0.83-2.11)	2.70 ± 0.88<br>(1.48-4.50)
VA-TA	1.29 ± 0.11<br>(1.07-1.52)	1.89 ± 0.12<br>(1.68-2.19)	2.03 ± 0.11<br>(1.88-2.29)	0.90 ± 0.14<br>(0.65-1.19)	2.44 ± 0.19<br>(1.95-2.76)	1.35 ± 0.19<br>(0.98-1.67)	1.66 ± 0.10<br>(1.49-1.86)	1.78 ± 0.61<br>(1.11-3.17)	1.40 ± 0.33<br>(0.79-2.16)	2.56 ± 0.65<br>(1.28-3.72)
VA-CE	1.34 ± 0.15<br>(1.11-1.69)	1.89 ± 0.14<br>(1.52-2.10)	2.03 ± 0.15<br>(1.72-2.30)	0.97 ± 0.14<br>(0.81-1.40)	2.53± 0.15<br>(2.32-2.95)	1.34 ± 0.15<br>(1.16-1.82)	1.66 ± 0.11<br>(1.38-1.87)	1.37 ± 0.33<br>(0.98-2.09)	1.18 ± 0.24<br>(0.85-1.76)	2.28 ± 0.62<br>(1.28-3.72)
VA-B/1	1.39 ± 0.18<br>(1.02-1.64)	1.61 ± 0.15<br>(1.39-1.91)	1.93 ± 0.24<br>(1.40-2.26)	0.52 ± 0.14<br>(0.32-0.78)	2.00 ± 0.32<br>(1.51-2.58)	1.25 ± 0.12<br>(1.07-1.47)	2.51 ± 0.34<br>(1.92-3.09)	2.49 ± 0.89<br>(1.32-4.88)	3.60 ± 1.75<br>(1.29-7.08)	2.64 ± 0.94<br>(1.62-5.84)
VA-B/2	1.34 ± 0.14<br>(1.16-1.64)	1.39 ± 0.16<br>(1.09-1.81)	1.71 ± 0.14<br>(1.43-2.10)	0.49 ± 0.06<br>(0.41-0.65)	1.76 ± 0.12<br>(1.50-2.08)	1.45 ± 0.12<br>(1.26-1.71)	2.09 ± 0.19<br>(1.72-2.52)	2.02 ± 0.67<br>(1.02-3.69)	1.89 ± 0.92<br>(0.89-4.44)	2.27 ± 0.63<br>(1.26-3.67)
VA-BS	1.43 ± 0.20<br>(1.11-1.77)	1.62 ± 0.11<br>(1.41-1.83)	2.07 ± 0.28<br>(1.56-2.51)	0.57 ± 0.15<br>(0.33-0.80)	2.02 ± 0.38<br>(1.45-2.58)	1.34 ± 0.15<br>(1.11-1.62)	2.15 ± 0.20<br>(1.72-2.44)	2.34 ± 0.72<br>(1.32-3.87)	2.81 ± 1.00<br>(1.58-4.89)	1.80 ± 0.29<br>(1.38-2.32)
VA-AZ	1.36 ± 0.09<br>(1.23-1.59)	1.79 ± 0.17<br>(1.49-2.23)	2.09 ± 0.12<br>(1.81-2.41)	0.71 ± 0.11<br>(0.53-0.92)	2.42 ± 0.17<br>(2.09-2.78)	0.97 ± 0.27<br>(0.51-1.31)	2.13 ± 0.25<br>(1.77-2.73)	1.98 ± 0.54<br>(1.20-3.03)	2.20 ± 0.43<br>(1.18-3.13)	1.89 ± 0.67<br>(1.15-3.65)
VA-SG	1.36 ± 0.09<br>(1.23-1.52)	1.65 ± 0.23<br>(1.32-2.12)	2.06 ± 0.24<br>(1.69-2.63)	0.60 ± 0.10<br>(0.41-0.74)	2.22 ± 0.27<br>(1.75-2.89)	1.21 ± 0.18<br>(0.89-1.72)	3.10 ± 0.29<br>(2.64-3.73)	3.32 ± 0.83<br>(1.70-4.55)	2.97 ± 0.95<br>(1.60-6.19)	2.18 ± 0.54<br>(1.35-3.59)
VK-BS	1.23 ± 0.10<br>(1.04-1.42)	1.66 ± 0.20<br>(1.34-1.94)	1.87 ± 0.19<br>(1.38-2.18)	0.93 ± 0.12<br>(0.74-1.22)	2.28 ± 0.16<br>(1.88-2.55)	1.57 ± 0.23<br>(0.72-1.83)	1.99 ± 0.25<br>(1.52-2.61)	1.24 ± 0.26<br>(0.96-1.88)	1.08 ± 0.24<br>(0.63-1.55)	2.67 ± 0.67<br>(1.64-4.22)
VK- NA	1.15 ± 0.06<br>(1.06-1.25)	1.60 ± 0.18<br>(1.28-1.90)	1.83 ± 0.11<br>(1.69-2.06)	0.88 ± 0.09<br>(0.69-1.05)	2.46 ± 0.14<br>(2.18-2.68)	1.20 ± 0.16<br>(0.93-1.51)	1.74 ± 0.12<br>(1.47-1.89)	2.30 ± 0.59<br>(1.43-3.08)	1.68 ± 0.34<br>(1.20-2.33)	2.10 ± 0..50<br>(1.48-3.02)
VK-NE	1.14 ± 0.12<br>(0.99-1.28)	1.96 ± 0.13<br>(1.75-2.11)	1.93 ± 0.14<br>(1.70-2.08)	0.82 ± 0.12<br>(0.72-1.04)	2.25 ± 0.07<br>(2.18-2.36)	1.28 ± 0.32<br>(0.99-1.82)	1.77 ± 0.12<br>(1.62-1.96)	3.94 ± 1.00<br>(2.28-5.31)	2.04 ± 0.25<br>(2.47-1.73)	1.50 ± 0.45<br>(1.14-2.37)
VT-PG	1.29 ± 0.08<br>(1.16-1.47)	1.54 ± 0.18<br>(1.25-1.97)	1.70 ± 0.16<br>(1.48-2.03)	0.39 ± 0.05<br>(0.31-0.48)	1.67 ± 0.14<br>(1.50-2.01)	1.33 ± 0.14<br>(1.02-1.59)	2.84 ± 0.46<br>(2.27-3.82)	2.35 ± 0.53<br>(1.60-3.55)	2.87 ± 1.22<br>(1.58-7.00)	2.01 ± 0.60<br>(1.04-3.56)
